# Association of Antihypertensives That Stimulate vs Inhibit Types 2 and 4 Angiotensin II Receptors With Cognitive Impairment

**DOI:** 10.1001/jamanetworkopen.2021.45319

**Published:** 2022-01-28

**Authors:** Zachary A. Marcum, Jordana B. Cohen, Chong Zhang, Catherine G. Derington, Tom H. Greene, Lama Ghazi, Jennifer S. Herrick, Jordan B. King, Alfred K. Cheung, Nick Bryan, Mark A. Supiano, Joshua A. Sonnen, William S. Weintraub, Jeff Williamson, Nicholas M. Pajewski, Adam P. Bress

**Affiliations:** 1 Department of Pharmacy, School of Pharmacy, University of Washington, Seattle; 2 Renal-Electrolyte and Hypertension Division, Department of Medicine, Perelman School of Medicine at the University of Pennsylvania, Philadelphia; 3 Department of Biostatistics, Epidemiology, and Informatics, Perelman School of Medicine, University of Pennsylvania, Philadelphia; 4 Division of Health System Innovation and Research, Department of Population Health Sciences, University of Utah School of Medicine, Salt Lake City; 5 Clinical and Translational Research Accelerator, Yale University School of Medicine, New Haven, Connecticut; 6 George E. Wahlen Department of Veterans Affairs Medical Center, Salt Lake City, Utah; 7 Institute for Health Research, Kaiser Permanente Colorado, Aurora; 8 Department of Internal Medicine, University of Utah School of Medicine, Salt Lake City; 9 Department of Radiology, Perelman School of Medicine, University of Pennsylvania, Philadelphia; 10 Division of Geriatrics, University of Utah School of Medicine, Salt Lake City; 11 Department of Pathology and Neurology and Neurosurgery, McGill University School of Medicine, Montreal, Quebec, Canada; 12 MedStar Health Research Institute and Georgetown University, Washington, DC; 13 Section on Gerontology and Geriatric Medicine, Wake Forest School of Medicine, Winston-Salem, North Carolina; 14 Department of Biostatistics and Data Science, Wake Forest School of Medicine, Winston-Salem, North Carolina

## Abstract

**Importance:**

Use of antihypertensive medications that stimulate type 2 and 4 angiotensin II receptors, compared with those that do not stimulate these receptors, has been associated with a lower risk of dementia. However, this association with cognitive outcomes in hypertension trials, with blood pressure levels in the range of current guidelines, has not been evaluated.

**Objective:**

To examine the association between use of exclusively antihypertensive medication regimens that stimulate vs inhibit type 2 and 4 angiotensin II receptors on mild cognitive impairment (MCI) or dementia.

**Design, Setting, and Participants:**

This cohort study is a secondary analysis (April 2011 to July 2018) of participants in the randomized Systolic Blood Pressure Intervention Trial (SPRINT), which recruited individuals 50 years or older with hypertension and increased cardiovascular risk but without a history of diabetes, stroke, or dementia. Data analysis was conducted from March 16 to July 6, 2021.

**Exposures:**

Prevalent use of angiotensin II receptor type 2 and 4–stimulating or –inhibiting antihypertensive medication regimens at the 6-month study visit.

**Main Outcomes and Measures:**

The primary outcome was a composite of adjudicated amnestic MCI or probable dementia.

**Results:**

Of the 8685 SPRINT participants who were prevalent users of antihypertensive medication regimens at the 6-month study visit (mean [SD] age, 67.7 [11.2] years; 5586 [64.3%] male; and 935 [10.8%] Hispanic, 2605 [30.0%] non-Hispanic Black, 4983 [57.4%] non-Hispanic White, and 162 [1.9%] who responded as other race or ethnicity), 2644 (30.4%) were users of exclusively stimulating, 1536 (17.7%) inhibiting, and 4505 (51.9%) mixed antihypertensive medication regimens. During a median of 4.8 years of follow-up (95% CI, 4.7-4.8 years), there were 45 vs 59 cases per 1000 person-years of amnestic MCI or probable dementia among prevalent users of regimens that contained exclusively stimulating vs inhibiting antihypertensive medications (hazard ratio [HR], 0.76; 95% CI, 0.66-0.87). When comparing stimulating-only vs inhibiting-only users, amnestic MCI occurred at rates of 40 vs 54 cases per 1000 person-years (HR, 0.74; 95% CI, 0.64-0.87) and probable dementia at rates of 8 vs 10 cases per 1000 person-years (HR, 0.80; 95% CI, 0.57-1.14). Negative control outcome analyses suggested the presence of residual confounding.

**Conclusions and Relevance:**

In this secondary analysis of SPRINT, prevalent users of regimens that contain exclusively antihypertensive medications that stimulate vs inhibit type 2 and 4 angiotensin II receptors had lower rates of incident cognitive impairment. Residual confounding cannot be ruled out. If these results are replicated in randomized clinical trials, certain antihypertensive medications could be prioritized to prevent cognitive decline.

## Key Points

**Question:**

Are antihypertensive medications that stimulate type 2 and 4 angiotensin II receptors, compared with those that do not stimulate these receptors, associated with a lower risk of incident cognitive impairment?

**Findings:**

In a secondary analysis of the Systolic Blood Pressure Intervention Trial (SPRINT), this cohort study of 8685 patients found that prevalent use of medication regimens that contain exclusively angiotensin II receptor type 2 and 4–stimulating antihypertensives was associated with an approximately 25% lower risk of incident amnestic mild cognitive impairment or probable dementia during 4.8 years of follow-up.

**Meaning:**

These results, if replicated in randomized clinical trials, suggest that certain antihypertensive medications could be used to prevent the development of cognitive decline.

## Teaser

This secondary analysis cohort study of Systolic Blood Pressure Intervention Trial (SPRINT) participants assesses the association between antihypertensive medications that stimulate type 2 and 4 angiotensin II receptors with the risk of incident cognitive impairment.

## Introduction

Hypertension, affecting nearly half of adults in the US, is a leading modifiable risk factor for dementia.^1,2,3^ Lower blood pressure (BP), especially in midlife, is associated with lower risk of dementia in observational studies.^1,2^ Recent trial evidence has shown that targeting intensive BP control using a variety of antihypertensive classes reduces mild cognitive impairment (MCI) and dementia risk even at older ages compared with standard BP control.^4^ Because there are no effective disease-modifying treatments for dementia, prevention through optimal hypertension control is critical to reducing the public health burden of dementia.^1^ However, it remains unclear whether certain classes of antihypertensive medications are more effective at preventing dementia than others, independent of their BP-lowering effects.

One observational analysis^5^ of 1909 community-dwelling older adults (mean age, 74.5 years) found that prevalent users of angiotensin II receptor type 2 and 4–stimulating antihypertensives had lower dementia rates compared with users of antihypertensives that inhibit the same receptors (eFigure 1 in the Supplement). The renin-angiotensin system is a biochemical pathway with a long-term and integral role in the pathogenesis of dementia, largely driven by angiotensin II.^6^ Animal and mechanistic data show that antihypertensive medications that stimulate type 2 and 4 angiotensin II receptors promote beneficial effects on the brain, possibly through reduced ischemia, enhanced cerebral blood flow, and improved spatial memory processing, among other pathways.^7,8,9,10,11,12,13,14,15,16^

Although use of antihypertensive medications that stimulate (angiotensin II receptor type 1 blockers, dihydropyridine calcium channel blockers, and thiazide diuretics) vs inhibit (angiotensin-converting enzyme [ACE] inhibitors, β-blockers, and nondihydropyridine calcium channel blockers) type 2 and 4 angiotensin II receptors has been associated with lower risk of dementia, their association with cognitive outcomes in hypertension trials, with BP levels in the range of current guidelines, has not been evaluated. Examining this question in the context of contemporary BP levels can provide clinically relevant insights into antihypertensive associations with adjudicated cognitive outcomes, independent of their BP-lowering effects. Thus, we assessed the association of prevalent use of antihypertensive regimens that exclusively contain medications that stimulate vs inhibit type 2 and 4 angiotensin II receptors on MCI or probable dementia in the Systolic Blood Pressure Intervention Trial (SPRINT).

## Methods

### Study Design

**Figure 1.**
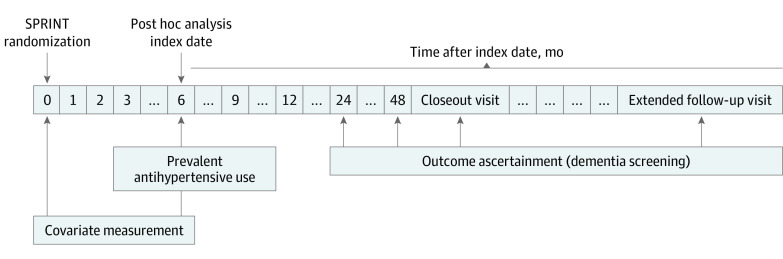
Study Design This is a prevalent-user study design, with the exposure of interest being antihypertensive use at the 6-month visit. Antihypertensive use was categorized into angiotensin II receptor type 2 and 4–stimulating (angiotensin II receptor blockers, dihydropyridine calcium channel blockers, and/or thiazides) and/or –inhibiting (angiotensin-converting enzyme inhibitors, β-blockers, and/or nondihydropyridine calcium channel blockers) antihypertensives. Three mutually exclusive antihypertensive use categories were created: stimulating only, inhibiting only, and mixed (use of both stimulating and inhibiting). Covariates were measured using data from Systolic Blood Pressure Intervention Trial (SPRINT) randomization as well as at the 6-month visit. Dementia screening was conducted at 24 and 48 months after SPRINT randomization as well as at the SPRINT closeout visit and an extended follow-up visit.

This cohort study is a secondary analysis of SPRINT. Figure 1 shows the timeline for assessment of antihypertensive medication use and study outcomes. The primary medication exposure was assessed at the 6-month study visit because most antihypertensive changes occurred in the first 3 months and had stabilized, on average, after the 6-month visit (eFigures 2 and 3 in the Supplement).^17^ Participants were followed up from the 6-month study visit until the occurrence of an outcome event or the end of active trial follow-up (April 2011 to July 2018). The trial was approved by the institutional review board at each participating site, and each participant provided written informed consent. All data were deidentified. Data analysis was conducted from March 16 to July 6, 2021. This report follows the Strengthening the Reporting of Observational Studies in Epidemiology (STROBE) reporting guideline.

### Study Participants and Design

The SPRINT design and cognitive results have been previously described.^18,19^ In the parent trial, participants were 50 years or older with systolic BP (SBP) between 130 and 180 mm Hg and had increased cardiovascular risk defined by having clinical or subclinical cardiovascular disease (CVD), chronic kidney disease (defined as an estimated glomerular filtration rate <60 mL/min/1.73 m^2^), a 10-year Framingham CVD risk of 15% or greater, or age of 75 years or older. Individuals were excluded if they were living in a nursing home, had a diagnosis of dementia, or were receiving medications primarily for dementia. Participants with diabetes or a history of stroke were also excluded. A total of 9361 participants were randomized between November 2010 and March 2013 in a 1:1 allocation to an intensive treatment strategy with an SBP goal of less than 120 mm Hg or a standard treatment strategy with an SBP goal of less than 140 mm Hg. Hypertension treatment algorithms and formulary are included in the trial protocol and briefly in eMethods 1 in the Supplement.^19^ The SPRINT treatment protocol was flexible in terms of choice and dose of antihypertensive medications, with preferences among the drug classes specified based on CVD outcome trials and current guidelines.

### Study Follow-up

The trial planned cognitive assessments at baseline and at 2 and 4 years of follow-up, as well as at study closeout (Figure 1). On August 20, 2015, the intervention was stopped. A final extended follow-up visit, conducted between October 2017 and July 2018, included cognitive assessment. For this analysis, the final date of follow-up was July 22, 2018. For both randomized groups, visit frequency was monthly for the first 3 months after randomization, then every 3 months for the duration of the trial. Additional visits were scheduled as needed for management of adverse effects or for monitoring significant medication changes or other clinical issues.

### Antihypertensive Use at the 6-Month Study Visit

For the primary analysis, participants from both trial treatment groups were pooled and classified into 1 of 2 mutually exclusive exposure categories according to their prevalent antihypertensive medication regimen at the 6-month visit (ie, the index date): (1) use of only angiotensin II receptor type 2 and 4–stimulating antihypertensives (angiotensin II receptor type 1 blockers, dihydropyridine calcium channel blockers, and thiazides) and (2) use of only angiotensin II receptor–inhibiting antihypertensives (ACE inhibitors, β-blockers, and nondihydropyridine calcium channel blockers). In secondary analysis, we also compared users of at least 1 antihypertensive medication from categories 1 and 2 (ie, mixed regimens) vs inhibiting-only users.

### Outcome Ascertainment and Adjudication

We selected a priori our primary outcome as a composite of adjudicated amnestic MCI or probable dementia. The definition of MCI in the original SPRINT protocol (ie, protocol-defined MCI) was time to the first of 2 consecutive occurrences of MCI (amnestic or nonamnestic). For this analysis, we chose a single occurrence of amnestic MCI as the primary definition for MCI. This decision was made to capture as many MCI events as possible, to avoid conditioning the outcome on future assessments, and because amnestic MCI has consistently been associated with increased risk of progression to dementia.^20,21,22,23,24,25,26,27,28,29,30,31^ Secondary outcomes included probable dementia or protocol-defined MCI, probable dementia alone, amnestic MCI alone, and protocol-defined MCI alone. In addition, to incorporate the competing risk of death, we added death as an additional event to each of the outcomes and on its own. Details of the cognitive outcome ascertainment process in the trial are provided elsewhere^4^ and in eMethods 2 and eFigure 4 in the Supplement.

### Covariates

Covariates used for adjustment were selected a priori based on their potential role as confounders. We determined sociodemographic characteristics, comorbid conditions, cognitive function, and concomitant nonantihypertensive medications at the randomization visit because this was the only available measurement before the index date (Figure 1). In addition, because vital signs and laboratory results were measured more frequently, we used the most recent value before the index date.

### Propensity Score Estimation

To adjust for measured confounding at baseline, we generated propensity scores to estimate the probability of being a prevalent user of an antihypertensive regimen that contained exclusively stimulating vs inhibiting medications using logistic regression with all baseline covariates included in the model. We assessed the overlap in the distribution of the propensity scores between medication user groups (eFigures 5 and 6 in the Supplement). We verified covariate balance in the samples using absolute standardized mean differences.

### Statistical Analysis

We compared baseline characteristics between prevalent users of exclusively stimulating vs inhibiting antihypertensive medication regimens before and after weighting. We used inverse probability of treatment–weighted Cox proportional hazards regression to calculate adjusted hazard ratios (HRs) for each outcome associated with the medication exposure. We applied a 2-sided α of .05 for all hypothesis tests, without correction for multiple comparisons. Missing values for covariates were imputed using the mean for continuous variables. Missing values were classified as a separate category for categorical variables if the prevalence of missingness was more than 3% (and deleted if the prevalence of missingness was <3%; n = 60 participants). The 95% CIs and *P* values for all analyses are based on bootstrap resampling.

All analyses, including the logistic regression models to estimate propensity scores, were repeated in subgroups by age, sex, race and ethnicity, history of CVD, chronic kidney disease (estimated glomerular filtration rate calculated using the Modification of Diet in Renal Disease Study equation^32^), body mass index (calculated as weight in kilograms divided by height in meters squared), number of antihypertensive medication classes, SBP tertile, and treatment group. Race and ethnic group were self-reported. Interaction 2-sided *P* values comparing exposure associations between subgroups were computed by Wald tests applied to the inverse probability of treatment estimates of exposure associations and their associated bootstrap SEs using separate propensity models within each subgroup. In sensitivity analyses, the Cox proportional hazards regression models were rerun using the same covariates but implemented via the following strategies: (1) unadjusted, (2) minimally adjusted (age, sex, and race and ethnicity), (3) multivariable adjusted, (4) propensity score as a covariate, (5) propensity score stratification (6 strata), (6) propensity score matching (1:1), and (7) matching weight adjusted.^33^ In secondary analyses, we repeated all analyses comparing prevalent users of mixed antihypertensive medication regimens (ie, using at least 1 stimulating and 1 inhibiting medication) with those exclusively using inhibiting medications. Finally, we estimated risk of residual confounding by examining 3 negative control outcomes (ie, outcomes thought not to be caused by the medication exposure groups).^34^ To identify negative control outcomes in SPRINT, we reviewed *Medical Dictionary for Regulatory Activities* codes for the serious adverse events deemed not to be related to the treatment. The conditions included separate composite infectious, orthopedic, and hematologic outcomes.

The primary analyses followed intention-to-treat principles (ie, postindex date variables, such as BP levels, were not incorporated into the analysis). All analyses were completed using R software, version 4.0.2 (R Foundation for Statistical Computing).

## Results

### Participant Characteristics

Of the 8685 SPRINT participants who were prevalent users of antihypertensive medication regimens at the 6-month study visit (mean [SD] age, 67.7 [11.2] years; 5586 [64.3%] male; and 935 [10.8%] Hispanic, 2605 [30.0%] non-Hispanic Black, 4983 [57.4%] non-Hispanic White, and 162 [1.9%] who responded as other race or ethnicity), 2644 (30.4%) were users of exclusively stimulating, 1536 (17.7%) inhibiting, and 4505 (51.9%) mixed antihypertensive medication regimens. Baseline characteristics of prevalent users of regimens that contained exclusively stimulating vs inhibiting antihypertensives are listed in Table 1. Among prevalent users of stimulating compared with inhibiting regimens, there were higher proportions of women (1023 [38.7%] vs 461 [30.0%]), Black participants (886 [33.5%] vs 301 [19.6%]), and participants randomized to intensive treatment (1246 [47.1%] vs 439 [28.6%]). Prevalent users of stimulating regimens also had a lower prevalence at baseline of a history of CVD (182 [6.9%] vs 372 [24.2%]), coronary revascularization (90 [3.4%] vs 231 [15.0%]), atrial fibrillation or flutter (108 [4.1%] vs 180 [11.7%]), and statin use (937 [35.4%] vs 777 [50.6%]) compared with users of inhibiting regimens. No substantial differences were found in baseline characteristics after weighting (all absolute standardized mean differences <0.1) (eFigure 7 in the Supplement). During study follow-up, SBP was also similar between the stimulating and inhibiting regimen user groups (eFigure 8 in the Supplement).

**Table 1.** Baseline Characteristics Between Prevalent Users of AT2R- and AT4R-Stimulating vs -Inhibiting Antihypertensives at the 6-Month Visit and Before and After Inverse Probability Weighting^a^

Characteristic^b^	Before weighting			After weighting		
	AT2R/AT4R		ASD	AT2R/AT4R		ASD
	Stimulating, No. (%) (n = 2644)	Inhibiting, No. (%) (n = 1536)		Stimulating, No. (%)	Inhibiting, No. (%)	
Demographic characteristics						
Age, mean (SD), y	67.2 (9.4)	68.6 (9.2)	0.15	67.8 (9.5)	68 (9.4)	0.02
Sex						
Female	1023 (38.7)	461 (30.0)	0.18	35.7	35.6	0
Male	1621 (61.3)	1075 (70.0)	NA	64.3	64.4	NA
Race and ethnicity						
Hispanic	319 (12.1)	171 (11.1)	0.03	12.0	12.3	0.01
Non-Hispanic Black	886 (33.5)	301 (19.6)	0.31	28.3	26.5	0.04
Non-Hispanic White	1383 (52.3)	1038 (67.6)	0.3	57.8	59.1	0.03
Social and behavioral						
Lives with others	1864 (70.5)	1096 (71.4)	0.02	70.5	70.2	0.01
Has private insurance	1217 (46.9)	608 (39.6)	0.13	44.5	44.2	0.01
Current smoker	363 (13.7)	179 (11.7)	0.06	12.8	12.3	0.01
Former smoker	1040 (39.3)	678 (44.1)	0.1	41.0	41.1	0
Never smoker	1239 (46.9)	677 (44.1)	0.06	46.3	46.6	0.01
Educational level						
Less than high school	216 (8.2)	164 (10.7)	0.08	8.6	9.1	0.01
High school graduate only	405 (15.3)	236 (15.4)	0.01	15.7	15.7	0
Post–high school graduate	959 (36.3)	511 (33.3)	0.06	34.9	33.9	0.02
College graduate or greater	1064 (40.2)	625 (40.7)	0.01	40.8	41.3	0.01
Health insurance status						
Medicare	1358 (51.4)	926 (60.3)	0.18	54.7	55.6	0.02
Medicaid	166 (6.3)	123 (8.0)	0.07	7.0	7.3	0.01
VA	443 (16.8)	333 (21.7)	0.13	17.9	18.1	0.01
Usual source of care						
Physician’s office or outpatient clinic	2235 (84.5)	1324 (86.2)	0.04	85.6	85.9	0.01
Community health care facility or other	292 (11.0)	148 (9.6)	0.04	10.3	10.2	0
No usual source of care	111 (4.2)	62 (4.0)	0.01	4.1	3.8	0.01
Medical history						
Clinical CVD	182 (6.9)	372 (24.2)	0.51	12.6	13.5	0.03
Left ventricular hypertrophy	457 (17.3)	215 (14.0)	0.09	16.1	15.0	0.03
Dizziness when standing	124 (4.7)	73 (4.8)	0	4.7	4.5	0
History of coronary revascularization	90 (3.4)	231 (15.0)	0.43	7.2	7.8	0.02
History of depression	462 (17.5)	296 (19.3)	0.05	17.9	18.0	0
History of atrial fibrillation or flutter	108 (4.1)	180 (11.7)	0.3	7.0	7.3	0.01
Baseline cognitive assessments						
Montreal Cognitive Assessment score, median (IQR)^c^	23.1 (4.0)	22.8 (4.2)	0.07	23.0 (4.0)	23.0 (4.2)	0
Logical Memory form II score, median (IQR)^d^	8.4 (3.3)	8.3 (3.3)	0.03	8.3 (3.3)	8.3 (3.3)	0
Digit Symbol Coding Test score, median (IQR)^e^	52.0 (15.9)	50.7 (15)	0.09	51.5 (15.8)	51.4 (15.2)	0.01
Clinical and laboratory measurements, mean (SD)						
SBP, mm Hg	139.7 (14.6)	135.8 (15.0)	0.26	138.7 (14.3)	138.5 (15.5)	0.01
DBP, mm Hg	79.4 (11.5)	76.1 (11.2)	0.28	78.2 (11.6)	77.9 (11.1)	0.03
Resting heart rate, beats/min	67.7 (11.2)	65.8 (11.4)	0.17	67.0 (11.3)	66.8 (11.4)	0.01
Serum potassium, mEq/L	4.1 (0.4)	4.3 (0.5)	0.46	4.2 (0.5)	4.2 (0.4)	0.05
Serum creatinine, mg/dL	1.0 (0.3)	1.1 (0.3)	0.17	1.1 (0.3)	1.1 (0.3)	0.02
Albumin to creatinine ratio, mg/g	32.1 (109.1)	42.8 (184.8)	0.08	38.8 (146.9)	38.4 (152.7)	0
Total cholesterol, mg/dL	194.9 (39.7)	184.3 (40.8)	0.26	191.5 (40.4)	191.0 (40.8)	0.01
HDL-C, mg/dL	54.3 (14.4)	51.6 (14.3)	0.19	53.5 (14.1)	53.5 (15.2)	0
Triglycerides, mg/dL	120.7 (96.9)	127.7 (77.1)	0.08	123.1 (106.3)	124.2 (73.6)	0.01
BMI	29.8 (5.6)	29.6 (5.7)	0.04	29.7 (5.5)	29.6 (5.9)	0.02
Serum glucose, mg/dL	98.0 (13.8)	98.4 (13.1)	0.03	98.2 (13.3)	98.2 (14.4)	0
Medication use						
Aspirin	1228 (46.4)	867 (56.4)	0.19	49.3	50.2	0.02
Statin	937 (35.4)	777 (50.6)	0.31	40.6	41.9	0.03
NSAID	893 (33.8)	614 (40.0)	0.13	34.8	35.4	0.01
No. of nonantihypertensive medications	3.3 (3.0)	4.1 (3.2)	0.26	3.5 (3.1)	3.6 (3.1)	0.03
Randomized to intensive treatment	1246 (47.1)	439 (28.6)	0.38	41.2	39.1	0.04

Abbreviations: ASD, absolute standardized difference; AT2R, angiotensin II receptor type 2; AT4R, angiotensin II receptor type 4; BMI, body mass index (calculated as weight in kilograms divided by height in meters squared); CVD, cardiovascular disease; DBP, diastolic blood pressure; HDL-C, high-density lipoprotein cholesterol; NA, not applicable; NSAID, nonsteroidal anti-inflammatory drug; SBP, systolic blood pressure; VA, Veterans Affairs.

SI conversion factors: To convert potassium to millimoles per liter, multiply by 1; creatinine to micromoles per liter, multiply by 88.4; total cholesterol and HDL-C to micromoles per liter, multiply by 0.0259; triglycerides to millimoles per liter, multiply by 0.0113; and glucose to millimoles per liter, multiply by 0.0555.

^a^ Data for before weighting are presented as number (percentage) of participants, and data for after weighting as percentage of participants unless otherwise indicated. The total numbers of patients in the post–inverse probability weighted columns were omitted because the numbers were slightly different as a result of the weighting. Both AT2R- and AT4R-stimulating antihypertensives were defined as use of angiotensin II receptor blockers, dihydropyridine calcium channel blockers, and/or thiazides. Both AT2R- and AT4R-inhibiting antihypertensives were defined as use of angiotensin-converting enzyme inhibitors, β-blockers, and/or nondihydropyridine calcium channel blockers.

^b^ Values were missing for the following (stimulating/inhibiting): lives with others = 0/2, smoking = 2/2, source of care = 6/2, left ventricular hypertrophy = 129/86, dizziness when standing = 3/3, depression = 4/2, atrial fibrillation or flutter = 5/4, Montreal Cognitive Assessment = 18/11, Logical Memory Delayed Recall = 20/14, Digit Symbol Coding = 24/17, resting heart rate = 0/1, serum potassium = 1/1, serum creatinine = 10/3, albumin to creatinine ratio = 132/75, total cholesterol = 5/4, HDL-C = 5/4, triglycerides = 5/4, BMI = 15/12, serum glucose = 5/4, and estimated glomerular filtration rate = 10/3.

^c^ Scores range from 0 to 30, with higher scores denoting better global cognitive function.

^d^ Subtest of the Wechsler Memory Scale to measure learning and memory skills. Scores range from 0 to 14, with higher scores denoting better cognitive function.

^e^ Subtest of the Wechsler Adult Intelligence Scale to measure processing speed. Scores range from 0 to 135, with higher scores denoting better cognitive function.

### Outcomes

**Figure 2.**
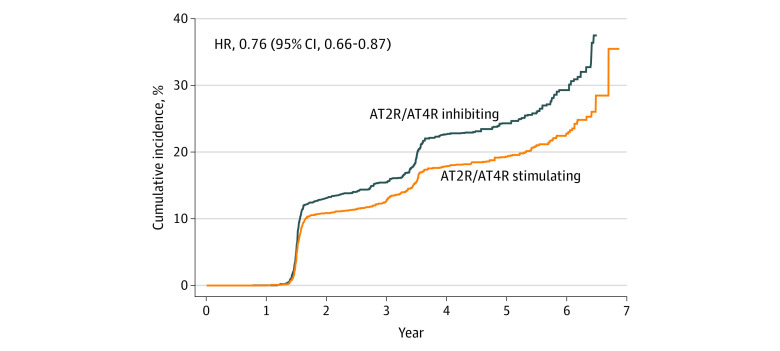
Cumulative Incidence Curves for Probable Dementia or Amnestic Mild Cognitive Impairment Cumulative incidence curves demonstrating the risk for probable dementia or amnestic mild cognitive impairment in Systolic Blood Pressure Intervention Trial participants between exposure groups were generated from Cox proportional hazards regression models using inverse probability treatment weighting. Participants are censored at death. Angiotensin II receptor type 2 (AT2R) and 4 (AT4R)–stimulating antihypertensives were defined as angiotensin II receptor blockers, dihydropyridine calcium channel blockers, and/or thiazides. Angiotensin II receptor type 2 and 4–inhibiting antihypertensives were defined as angiotensin-converting enzyme inhibitors, β-blockers, and/or nondihydropyridine calcium channel blockers. We omitted the number at risk over time because this model was generated using weighted samples. HR indicates hazard ratio.

During a median of 4.8 years of follow-up (95% CI, 4.7-4.8 years), there were 45 vs 59 cases per 1000 person-years of amnestic MCI or probable dementia among prevalent users of regimens that contained exclusively stimulating vs inhibiting antihypertensives (HR, 0.76; 95% CI, 0.66-0.87; n = 783 total events) (Table 2 and Figure 2). The secondary outcome of amnestic MCI alone (n = 685 total events) occurred at rates of 40 vs 54 cases per 1000 person-years among stimulating vs inhibiting users (HR, 0.74; 95% CI, 0.64-0.87). The secondary outcome of probable dementia alone (n = 140 total events) occurred at rates of 8 vs 10 cases per 1000 person-years among stimulating vs inhibiting users (HR, 0.80; 95% CI, 0.57-1.14) (Table 2 and eFigure 9 in the Supplement). The other secondary outcomes (protocol-defined MCI alone and probable dementia or protocol-defined MCI) are listed in Table 2 and eFigure 8 in the Supplement. Results when analyzing each study outcome as a composite with death are also given in Table 2 and eFigure 10 in the Supplement.

**Table 2.** Inverse Probability of Treatment Weighting–Adjusted Incidence Rates and HRs for Primary and Secondary Outcomes^a^

Outcome^b^	No. of events (events per 1000 person-years)		AT2R/AT4R-stimulating vs -inhibiting antihypertensive use weighted HR (95% CI)
	AT2R/AT4R-stimulating antihypertensive-only users (n = 2644)	AT2R/AT4R-inhibiting antihypertensive-only users (n = 1536)	
Primary outcome (censoring death)			
Probable dementia or amnestic MCI	428 (45)	355 (59)	0.76 (0.66-0.87)
Secondary outcomes (censoring death)			
Probable dementia alone	73 (8)	67 (10)	0.80 (0.57-1.14)
Amnestic MCI alone	373 (40)	312 (54)	0.74 (0.64-0.87)
Protocol-defined MCI alone	169 (17)	141 (22)	0.79 (0.63-1.00)
Probable dementia or protocol-defined MCI	222 (23)	183 (28)	0.81 (0.67-0.99)
Composite outcome (incorporating death)			
Probable dementia or amnestic MCI or death	502 (53)	428 (70)	0.75 (0.66-0.85)
Probable dementia or death	158 (16)	150 (21)	0.77 (0.60-0.98)
Amnestic MCI or death	452 (47)	389 (64)	0.73 (0.64-0.84)
Protocol-defined MCI or death	256 (26)	226 (34)	0.77 (0.64-0.93)
Probable dementia or protocol-defined MCI or death	304 (32)	264 (40)	0.79 (0.67-0.93)
Death	90 (12)	90 (16)	0.73 (0.52-1.02)

Abbreviations: AT2R, angiotensin II receptor type 2; AT4R, angiotensin II receptor type 4; HR, hazard ratio; MCI, mild cognitive impairment.

^a^ Angiotensin II receptor type 2 and 4–stimulating antihypertensives were defined as use of angiotensin II receptor blockers, dihydropyridine calcium channel blockers, and/or thiazides. Angiotensin II receptor type 2 and 4–inhibiting antihypertensives were defined as use of angiotensin-converting enzyme inhibitors, β-blockers, and/or nondihydropyridine calcium channel blockers.

^b^ For the primary and secondary outcomes, death was treated as a censoring event to produce cause-specific hazards. In addition, death was examined as a composite outcome with each of these outcomes and on its own.

### Subgroup, Sensitivity, and Secondary Analyses

Results were consistent across subgroups by age, sex, race and ethnicity, CVD, chronic kidney disease, body mass index, number of antihypertensive medication classes, SBP, and SPRINT treatment group (Table 3). Results were consistent for sensitivity analyses using different covariate adjustment strategies (Table 3).

**Table 3.** Incidence Rates and HRs Comparing the Association Between AT2R- and AT4R-Stimulating-Only vs Inhibiting-Only Antihypertensive Users and Probable Dementia or Amnestic Mild Cognitive Impairment by Covariate Adjustment Strategy and Among Subgroups^a^

Method	No. in model	No. of events (events per 1000 person-years)		AT2R/AT4R-stimulating vs -inhibiting antihypertensive use HR (95% CI)	*P* value for interaction
		AT2R/AT4R-stimulating antihypertensive-only users	AT2R/AT4R-inhibiting antihypertensive-only users		
**Covariate adjustment strategy**					
IPW adjusted (primary analysis)	4149	428 (45)	355 (59)	0.76 (0.66-0.87)	NA
Unadjusted	4149	428 (41)	355 (62)	0.66 (0.57-0.76)	NA
Minimally adjusted (age, sex, and race and ethnicity)	4149	428 (41)	355 (62)	0.64 (0.55-0.74)	NA
Multivariable adjusted	4149	428 (41)	355 (62)	0.71 (0.60-0.84)	NA
Propensity score					
As covariate	4149	428 (41)	355 (62)	0.75 (0.65-0.87)	NA
Stratification	4149	428 (45)	355 (59)	0.75 (0.65-0.86)	NA
Matching	3044	213 (35)	355 (62)	0.56 (0.45-0.70)	NA
Matching weight adjusted^b^	4149	428 (47)	355 (62)	0.76 (0.66-0.87)	NA
**Subgroup analyses**					
Age, y					
<75	3026	232 (31)	168 (39)	0.80 (0.65-0.99)	.78
≥75	1123	196 (92)	187 (120)	0.77 (0.63-0.93)	
Sex					
Female	1475	149 (38)	101 (55)	0.67 (0.53-0.85)	.22
Male	2674	279 (49)	254 (62)	0.80 (0.67-0.95)	
Race and ethnicity					
Hispanic	489	67 (60)	55 (84)	0.70 (0.48-1.00)	.81
Non-Hispanic Black	1174	161 (51)	79 (63)	0.81 (0.61-1.06)	
Non-Hispanic White	2405	193 (38)	213 (51)	0.75 (0.62-0.91)	
Clinical cardiovascular disease					
Yes	547	41 (72)	79 (69)	1.06 (0.71-1.59)	.08
No	3602	387 (42)	276 (58)	0.72 (0.62-0.83)	
Kidney impairment: estimated GFR, mL/min/1.73 m^2^					
<60	1035	118 (55)	121 (77)	0.71 (0.55-0.92)	.48
≥60	3114	310 (41)	234 (52)	0.79 (0.67-0.94)	
BMI					
<25	758	99 (62)	94 (82)	0.77 (0.57-1.00)	.64
25 to <30	1642	171 (44)	139 (61)	0.71 (0.57-0.89)	
≥30	1749	158 (39)	122 (47)	0.83 (0.65-1.07)	
No. of antihypertensive medications					
1	1879	169 (45)	208 (58)	0.76 (0.63-0.91)	.62
2	1461	156 (46)	103 (55)	0.83 (0.63-1.10)	
3	644	84 (48)	37 (85)	0.56 (0.33-0.94)	
Systolic blood pressure, by tertile					
1 (84-132 mm Hg)	1475	133 (41)	127 (56)	0.72 (0.56-0.92)	.60
2 (133-143 mm Hg)	1295	149 (50)	119 (66)	0.74 (0.59-0.94)	
3 (144-214 mm Hg)	1379	146 (44)	109 (52)	0.85 (0.67-1.09)	
SPRINT randomization arm					
Intensive arm	1675	197 (44)	98 (52)	0.85 (0.67-1.08)	.31
Standard arm	2474	231 (46)	257 (62)	0.73 (0.61-0.87)	

Abbreviations: AT2R, angiotensin II receptor type 2; AT4R, angiotensin II receptor type 4; BMI, body mass index (calculated as weight in kilograms divided by height in meters squared); GFR, glomerular filtration rate; HR, hazard ratio; IPW, inverse probability weighted; NA, not applicable; SPRINT, Systolic Blood Pressure Intervention Trial.

^a^ Angiotensin II receptor type 2 and 4–stimulating antihypertensives were defined as use of angiotensin II receptor blockers, dihydropyridine calcium channel blockers, and/or thiazides. Angiotensin II receptor type 2 and 4–inhibiting antihypertensives were defined as use of angiotensin-converting enzyme inhibitors, β-blockers, and/or nondihydropyridine calcium channel blockers.

^b^ The same propensity score model from primary analysis was used to estimate matching weights, which provides a weighted analog to 1:1 paired propensity score matching.

In secondary analyses that compared prevalent users of mixed antihypertensive medication regimens with those that exclusively used inhibiting medications, baseline characteristics followed similar patterns to the primary analysis (eTable 1 in the Supplement). No differences were found in baseline characteristics after weighting (all absolute standardized mean differences <0.1) (eFigure 11 in the Supplement). During study follow-up, SBP was lower among mixed vs inhibiting users (eFigure 12 in the Supplement). Overall, results of the primary and secondary outcomes were not qualitatively different compared with the primary analysis (eTables 2 and 3 and eFigure 13 in the Supplement). Moreover, an association was found between prevalent users of regimens that contained exclusively stimulating vs inhibiting (primary analysis) and mixed vs inhibiting (secondary analysis) antihypertensives with the negative control outcomes (eTables 4 and 5 in the Supplement). For example, prevalent users of mixed antihypertensive medication regimens had lower rates of the composite hematologic outcomes compared with those exclusively using inhibiting medications (HR, 0.57; 95% CI, 0.37-0.88). Finally, the prevalence of stimulating, inhibiting, or mixed antihypertensive medication regimen classification did not materially change between SPRINT randomization and the 6-month visit (eTable 6 in the Supplement).

## Discussion

In this cohort study, a secondary analysis of SPRINT, prevalent use of medication regimens that contained exclusively angiotensin II receptor type 2 and 4–stimulating antihypertensives (ie, angiotensin II receptor type 1 blockers, dihydropyridine calcium channel blockers, and thiazide diuretics) vs inhibiting (ie, ACE inhibitors, β-blockers, and nondihydropyridine calcium channel blockers) was associated with a 24% lower risk of incident amnestic MCI or probable dementia during 4.8 years of follow-up. Prevalent use of stimulating vs inhibiting regimens was also associated with a significant 26% lower risk of amnestic MCI alone and a nonsignificant 20% lower risk of probable dementia alone. Results were consistent when incorporating the competing risk of death and were independent of SBP, cardiovascular risk factors, sociodemographic characteristics, and baseline cognitive function. Results were robust to sensitivity and secondary analyses; however, negative control outcome analyses suggested the presence of unmeasured confounding.

Given the high prevalence of hypertension, even a small reduction in dementia risk achieved by prescribing certain antihypertensives could have a measurable effect on the overall burden of dementia. Reports from the National Academies of Sciences, Engineering, and Medicine^2^ and the *Lancet* Commission^1^ highlighted the importance of managing hypertension as a dementia risk reduction strategy. However, there is currently insufficient evidence to recommend prescribing certain classes of antihypertensives for dementia risk reduction. Accordingly, the National Academies’ report identified the comparative effectiveness of different classes of antihypertensives as a high priority for future research.^2^ To date, clinical trials of antihypertensive treatment strategies examining cognitive outcomes have tested BP-specific targets using a broad drug formulary^4,35^ or the effect of individual antihypertensives (eg, candesartan [stimulating] vs lisinopril [inhibiting]) in older adults with MCI.^36^ A clinical trial to test the hypothesis assessed in our study for primary prevention would take years to complete. Alternatively, observational studies in larger samples, using a new-user design, with validated cognitive outcomes could provide a useful replication. At the same time, these results may warrant clinical trials that test exclusive use of angiotensin II receptor type 2 and 4–stimulating antihypertensives vs nonstimulating regimens. The trial structure could be similar to the Antihypertensive and Lipid-Lowering Treatment to Prevent Heart Attack Trial (ALLHAT), which compared amlodipine vs chlorthalidone and lisinopril vs chlorthalidone,^37^ or the Study on Cognition and Prognosis in the Elderly (SCOPE), which compared a candesartan-based regimen with other antihypertensive regimens that did not contain candesartan.^38^

The results of the current analysis extend previous work using data from the Prevention of Dementia by Intensive Vascular Care (PreDIVA) trial,^5^ which found that prevalent users of only stimulating antihypertensives had a 45% lower risk of incident dementia compared with users of only inhibiting antihypertensives during 6.7 years of follow-up. Moreover, we build on previous work by showing a positive association of stimulating medications with lower rates of amnestic MCI, characterized by subjective memory impairment or informant report as well as objective memory impairment.^39^ Amnestic MCI has been consistently shown to be associated with progression to dementia.^20,21,22,23,24,25,26,27,28,29,30,31^ Taken together, these findings from observational data provide support for antihypertensive repurposing (ie, using antihypertensive medications to reduce dementia risk beyond their BP-lowering effects). On a population level, shifting antihypertensive prescribing from inhibiting to stimulating regimens, while adhering to current hypertension guideline recommendations, could be a promising strategy to reduce the burden of dementia. This strategy would mean shifting the treatment paradigm from ACE inhibitors to angiotensin II receptor type 1 blockers and reducing the amount of inappropriate β-blocker use in the absence of coronary heart disease or heart failure with reduced ejection fraction, which was estimated to be prescribed as initial monotherapy for hypertension in up to 20% of older adults without compelling indication (ie, coronary artery disease, systolic heart failure, or atrial fibrillation).^40^

Multiple animal and human studies^41,42,43,44,45,46,47,48,49,50,51,52^ help explain possible underlying mechanisms for our results. A sizable volume of animal model^41,42,43,44,45,46,47,48^ and human studies^49,50,51,52^ support the overarching hypothesis that beyond effects on BP, antihypertensive drugs that increase activity at the angiotensin type 2 and 4 receptors provide greater brain protection compared with those that decrease activity. Numerous studies^10,53,54,55^ suggest a role for angiotensin II and angiotensin IV activity in protecting against ischemia or enhancing cerebral blood flow, especially via activity at angiotensin type 2 and, possibly, type 4 receptors. Agonism at the angiotensin type 4 receptor may improve spatial memory processing.^15,16,45,55^ However, it is important to recognize that unresolved complexity remains in understanding the interaction among antihypertensives, the renin angiotensin system, and cognitive outcomes.^56^ For example, data from human brain tissue of decedents show that domain-specific (C- and N-domain) changes in ACE1 in Alzheimer disease could promote angiotensin II–mediated disease progression.^57^ However, more evidence is needed on how different ACE inhibitors interact with the 2 ACE1 domains. Translating this mechanistic work to clinical practice using existing antihypertensives is an area of active research.^6^

### Limitations

This study has several limitations. First, SPRINT did not enroll persons with type 2 diabetes, previous stroke, advanced kidney disease, or symptomatic heart failure, so our results should be interpreted accordingly. Second, we understand that a new-user design is ideal for estimating medication effects outside trials. However, the study question necessitated a prevalent-user design, which could introduce bias. Our active-comparator design and methods of covariate adjustment appeared to mitigate these sources of bias, as suggested by the balance achieved between exposure groups in our measured covariates after weighting. Unfortunately, our covariates were generally not measured at the time of antihypertensive initiation, which occurred before the SPRINT baseline visit for most patients. Hence, we cannot assess whether our weighted analyses succeeding in balancing prognostic patient characteristics at the exact time of treatment initiation. Both underadjustment caused by unmeasured confounding and overadjustment caused by inclusion of covariates measured after treatment initiation, which may be intermediate on the causal pathway between treatment and outcome, are possible. Some reassurance that our findings are not an artifact of overadjustment is provided by the consistency of our results in a minimally adjusted model that included only age, race and ethnicity, and sex as covariates. However, the results of the negative control analyses suggested the presence of residual confounding, which is common with a prevalent-user design.

## Conclusions

In SPRINT, prevalent users of regimens that contain exclusively antihypertensives that stimulate vs inhibit type 2 and 4 angiotensin II receptors had lower rates of cognitive impairment. The possibility of residual confounding cannot be ruled out. If these results are replicated in randomized clinical trials, certain antihypertensives could be prioritized to prevent the development of cognitive decline.

## Notes

Supplement. **eFigure 1.** Antihypertensive Activity on the Renin-Angiotensin System **eMethods 1.** SPRINT Treatment Protocol Summary **eFigure 2.** Modified Therapeutic Intensity Score Across SPRINT Follow Up, by Angiotensin II Receptor Type 2 and 4-Stimulating-Only and Inhibiting-Only Antihypertensive Use **eFigure 3.** Modified Therapeutic Intensity Score Across SPRINT Follow Up, by Angiotensin II Receptor Type 2 and 4-Stimulating and Inhibiting Antihypertensive (“Mixed”) Use and Inhibiting-Only Antihypertensive Use **eMethods 2.** Assessment and Adjudication of Cognitive Outcomes in SPRINT **eFigure 4.** Timeline for Follow-Up Cognitive Assessments in SPRINT **eFigure 5.** Distribution of Propensity Scores, Angiotensin II Receptor Type 2 and 4-Stimulating-Only and Inhibiting-Only Antihypertensive Use at 6-Month Visit **eFigure 6.** Distribution of Propensity Scores, Angiotensin II Receptor Type 2 and 4-Stimulating and Inhibiting Antihypertensive (“Mixed”) vs Inhibiting-Only Antihypertensive Use at 6-Month Visit **eFigure 7.** Absolute Standardized Differences Comparing Angiotensin II Receptor Type 2 and 4-Stimulating-Only and Inhibiting-Only Antihypertensive Use at 6-Month Visit, Unadjusted and Adjusted for Average Treatment Effect **eFigure 8.** Systolic Blood Pressure Across SPRINT Follow-Up, by Angiotensin II Receptor Type 2 and 4-Stimulating-Only and Inhibiting-Only Antihypertensive Use **eFigure 9.** Cumulative Incidence Curves for Secondary Outcomes, Angiotensin II Receptor Type 2 and 4-Stimulating-Only vs Inhibiting-Only Antihypertensive Users **eFigure 10.** Cumulative Incidence Curves for Secondary Outcomes With Death as a Composite, Angiotensin II Receptor Type 2 and 4-Stimulating-Only vs Inhibiting-Only Antihypertensive Users **eTable 1.** Baseline Characteristics Between Prevalent Users of Angiotensin II Receptor Type 2 and 4-Stimulating and Inhibiting Antihypertensives (“Mixed”) vs Inhibiting-Only at 6-Month Visit, Before and After Inverse Probability Weighting **eFigure 11.** Absolute Standardized Differences Comparing Angiotensin II Receptor Type 2 And 4-Stimulating and Inhibiting (“Mixed) vs Inhibiting-Only Antihypertensive Use at 6-Month Visit, Unadjusted and Adjusted for Average Treatment Effect **eFigure 12.** Systolic Blood Pressure Across SPRINT Follow-Up, by Angiotensin II Receptor Type 2 and 4-Stimulating And Inhibiting (“Mixed”) And Inhibiting-Only Antihypertensive Use **eTable 2.** Inverse Probability Treatment Weighting-Adjusted Incidence Rates and Hazard Ratios for Primary and Secondary Outcomes, Angiotensin II Receptor Type 2 and 4-Stimulating and Inhibiting (“Mixed”) vs Inhibiting-Only Antihypertensive Users **eTable 3.** Incidence Rates and Hazard Ratios Comparing the Association Between Angiotensin II Receptor Type 2 and 4-Stimulating and Inhibiting (“Mixed”) vs Inhibiting-Only Antihypertensive Users and Probable Dementia or Amnestic Mild Cognitive Impairment, by Covariate Adjustment Strategy and Among Sub-Groups **eFigure 13.** Cumulative Incidence Curves for Probable Dementia or Amnestic Mild Cognitive Impairment, Angiotensin II Receptor Type 2 and 4-Stimulating and Inhibiting (“Mixed”) vs Inhibiting-Only Antihypertensive Users **eTable 4.** Inverse Probability Treatment Weighting-Adjusted Incidence Rates and Hazard Ratios for Negative Control Outcomes, Angiotensin II Receptor Type 2 and 4-Stimulating-Only vs Inhibiting-Only Antihypertensive Use **eTable 5.** Inverse Probability Treatment Weighting-Adjusted Incidence Rates and Hazard Ratios for Negative Control Outcomes, Angiotensin II Receptor Type 2 and 4-Stimulating and Inhibiting (“Mixed”) vs Inhibiting-Only Antihypertensive Use **eTable 6.** Antihypertensive Sub-Class Use at SPRINT Baseline and 6-Month Visit, Defined by Angiotensin II Receptor Type 2 and 4-Stimulating and Inhibiting Classification at 6-Month Visit
