# Supplementary material for: Association of Antihypertensives That Stimulate vs Inhibit Types 2 and 4 Angiotensin II Receptors With Cognitive Impairment
Source: JAMA Netw Open. 2022 Jan 28;5(1):e2145319. doi: 10.1001/jamanetworkopen.2021.45319 (PMC8800076; doi:10.1001/jamanetworkopen.2021.45319)
Supplement: Supplement. — eFigure 1. Antihypertensive Activity on the Renin-Angiotensin System eMethods 1. SPRINT Treatment Protocol Summary eFigure 2. Modified Therapeutic Intensity Score Across SPRINT Follow Up, by Angiotensin II Receptor Type 2 and 4-Stimulating-Only and Inhibiting-Only Antihypertensive Use eFigure 3. Modified Therapeutic Intensity Score Across SPRINT Follow Up, by Angiotensin II Receptor Type 2 and 4-Stimulating and Inhibiting Antihypertensive (“Mixed”) Use and Inhibiting-Only Antihypertensive Use eMethods 2. Assessment and Adjudication of Cognitive Outcomes in SPRINT eFigure 4. Timeline for Follow-Up Cognitive Assessments in SPRINT eFigure 5. Distribution of Propensity Scores, Angiotensin II Receptor Type 2 and 4-Stimulating-Only and Inhibiting-Only Antihypertensive Use at 6-Month Visit eFigure 6. Distribution of Propensity Scores, Angiotensin II Receptor Type 2 and 4-Stimulating and Inhibiting Antihypertensive (“Mixed”) vs Inhibiting-Only Antihypertensive Use at 6-Month Visit eFigure 7. Absolute Standardized Differences Comparing Angiotensin II Receptor Type 2 and 4-Stimulating-Only and Inhibiting-Only Antihypertensive Use at 6-Month Visit, Unadjusted and Adjusted for Average Treatment Effect eFigure 8. Systolic Blood Pressure Across SPRINT Follow-Up, by Angiotensin II Receptor Type 2 and 4-Stimulating-Only and Inhibiting-Only Antihypertensive Use eFigure 9. Cumulative Incidence Curves for Secondary Outcomes, Angiotensin II Receptor Type 2 and 4-Stimulating-Only vs Inhibiting-Only Antihypertensive Users eFigure 10. Cumulative Incidence Curves for Secondary Outcomes With Death as a Composite, Angiotensin II Receptor Type 2 and 4-Stimulating-Only vs Inhibiting-Only Antihypertensive Users eTable 1. Baseline Characteristics Between Prevalent Users of Angiotensin II Receptor Type 2 and 4-Stimulating and Inhibiting Antihypertensives (“Mixed”) vs Inhibiting-Only at 6-Month Visit, Before and After Inverse Probability Weighting eFigure 11. Absolute Standardized Differences Comp [file jamanetwopen-e2145319-s001.pdf]

Supplementary Online Content

Marcum ZA, Cohen JB, Zhang C, et al; Systolic Blood Pressure Intervention Trial (SPRINT) Research Group. Association of antihypertensives that stimulate vs inhibit types 2 and 4 angiotensin II receptors with cognitive impairment. *JAMA Netw Open*. 2022;5(1):e2145319. doi:10.1001/jamanetworkopen.2021.45319

eFigure 1. Antihypertensive Activity on the Renin-Angiotensin System

eMethods 1. SPRINT Treatment Protocol Summary

eFigure 2. Modified Therapeutic Intensity Score Across SPRINT Follow Up, by Angiotensin II Receptor Type 2 and 4-Stimulating Only and Inhibiting Only Antihypertensive Use

eFigure 3. Modified Therapeutic Intensity Score Across SPRINT Follow Up, by Angiotensin II Receptor Type 2 and 4-Stimulating and Inhibiting Antihypertensive (“Mixed”) Use and Inhibiting Only Antihypertensive Use

eMethods 2. Assessment and Adjudication of Cognitive Outcomes in SPRINT

eFigure 4. Timeline for Follow-Up Cognitive Assessments in SPRINT

eFigure 5. Distribution of Propensity Scores, Angiotensin II Receptor Type 2 and 4-Stimulating Only and Inhibiting Only Antihypertensive Use at 6-Month Visit

eFigure 6. Distribution of Propensity Scores, Angiotensin II Receptor Type 2 and 4-Stimulating and Inhibiting Antihypertensive (“Mixed”) vs Inhibiting Only Antihypertensive Use at 6-Month Visit

eFigure 7. Absolute Standardized Differences Comparing Angiotensin II Receptor Type 2 and 4-Stimulating Only and Inhibiting Only Antihypertensive Use at 6-Month Visit, Unadjusted and Adjusted for Average Treatment Effect

eFigure 8. Systolic Blood Pressure Across SPRINT Follow-Up, by Angiotensin II Receptor Type 2 and 4-Stimulating Only and Inhibiting Only Antihypertensive Use

eFigure 9. Cumulative Incidence Curves for Secondary Outcomes, Angiotensin II Receptor Type 2 and 4-Stimulating Only vs Inhibiting Only Antihypertensive Users

eFigure 10. Cumulative Incidence Curves for Secondary Outcomes With Death as a Composite, Angiotensin II Receptor Type 2 and 4-Stimulating Only vs Inhibiting Only Antihypertensive Users

eTable 1. Baseline Characteristics Between Prevalent Users of Angiotensin II Receptor Type 2 and 4-Stimulating and Inhibiting Antihypertensives (“Mixed”) vs Inhibiting Only at 6-Month Visit, Before and After Inverse Probability Weighting

eFigure 11. Absolute Standardized Differences Comparing Angiotensin II Receptor Type 2 And 4-Stimulating and Inhibiting (“Mixed) vs Inhibiting Only Antihypertensive Use at 6-Month Visit, Unadjusted and Adjusted for Average Treatment Effect

eFigure 12. Systolic Blood Pressure Across SPRINT Follow-Up, by Angiotensin II Receptor Type 2 and 4-Stimulating And Inhibiting (“Mixed”) And Inhibiting Only Antihypertensive Use

eTable 2. Inverse Probability Treatment Weighting-Adjusted Incidence Rates and Hazard Ratios for Primary and Secondary Outcomes, Angiotensin II Receptor Type 2 and 4-Stimulating and Inhibiting (“Mixed”) vs Inhibiting Only Antihypertensive Users

eTable 3. Incidence Rates and Hazard Ratios Comparing the Association Between Angiotensin II Receptor Type 2 and 4-Stimulating and Inhibiting (“Mixed”) vs Inhibiting Only Antihypertensive Users and Probable Dementia or Amnestic Mild Cognitive Impairment, by Covariate Adjustment Strategy and Among Sub-Groups

eFigure 13. Cumulative Incidence Curves for Probable Dementia or Amnestic Mild Cognitive Impairment, Angiotensin II Receptor Type 2 and 4-Stimulating and Inhibiting (“Mixed”) vs Inhibiting Only Antihypertensive Users

eTable 4. Inverse Probability Treatment Weighting-Adjusted Incidence Rates and Hazard Ratios for Negative Control Outcomes, Angiotensin II Receptor Type 2 and 4-Stimulating Only vs Inhibiting Only Antihypertensive Use

eTable 5. Inverse Probability Treatment Weighting-Adjusted Incidence Rates and Hazard Ratios for Negative Control Outcomes, Angiotensin II Receptor Type 2 and 4-Stimulating And Inhibiting (“Mixed”) vs Inhibiting Only Antihypertensive Use

eTable 6. Antihypertensive Sub-Class Use at SPRINT Baseline and 6-Month Visit, Defined by Angiotensin II Receptor Type 2 and 4-Stimulating and Inhibiting Classification at 6-Month Visit

This supplementary material has been provided by the authors to give readers additional information about their work.

© 2022 Marcum ZA et al. *JAMA Network Open.*

eFigure 1. Antihypertensive activity on the renin-angiotensin system

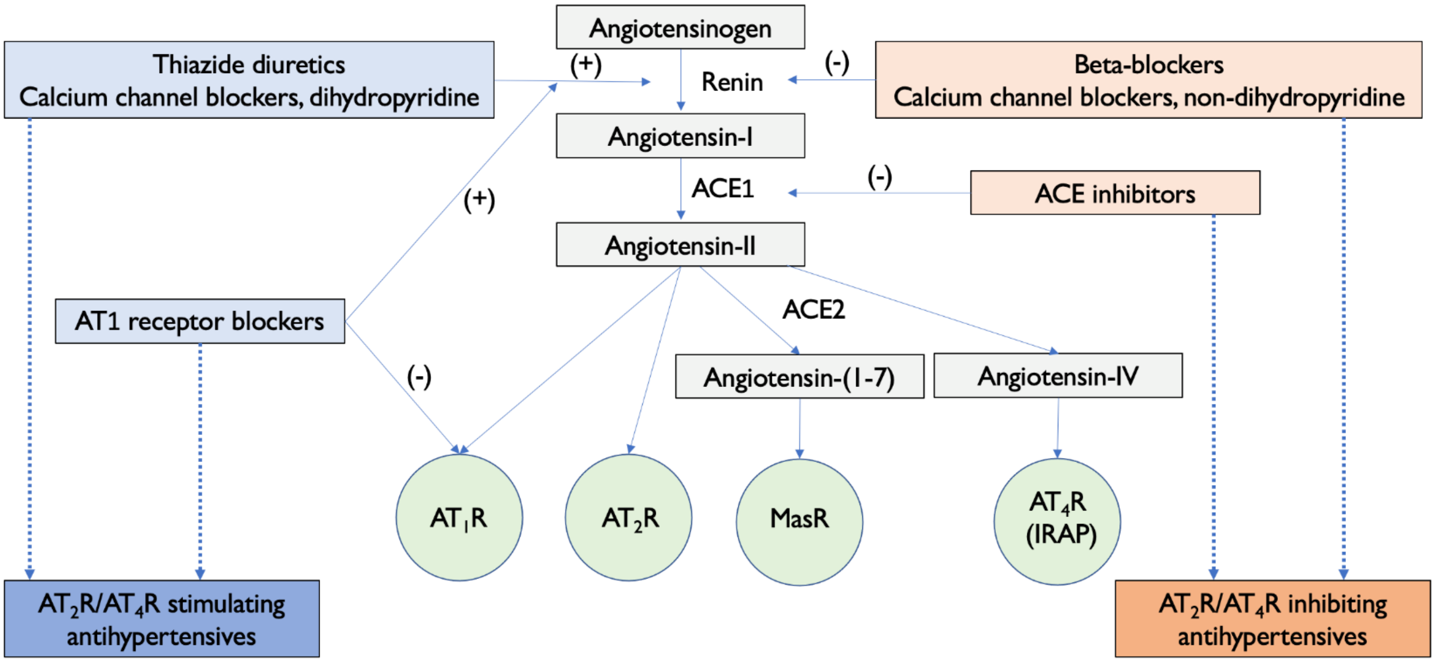

Thiazides and dihydropyridine calcium channel blockers (DiCCBs) increase renin. β–blockers (BB) reduce β1-mediated renin production. Long-acting forms of verapamil and diltiazem (non-DiCCBs) either do not affect or reduce renin. Renin generates angiotensin I, which is converted into angiotensin II by angiotensin-converting enzyme (ACE1), which exerts physiologic effects by binding to AT1R or AT2R or may be further metabolized into Angiotensin-IV, which binds to AT4R (IRAP). ACE inhibitors (ACEI) directly inhibit ACE1 activity, thereby inhibiting angiotensin II production. Angiotensin receptor 1 blockers (ARBs) inhibit angiotensin II activity directly at the AT1R but leave angiotensin II production intact. ACE1 reportedly degrades β-amyloid (Aβ), a major component of the cerebral neuritic plaques associated with Alzheimer's disease. ACEIs may inhibit this degradation, thus facilitating Aβ plaque accumulation. ARBs selectively inhibit Angiotensin-II at AT1R without inhibiting ACE1, allowing ACE1 to degrade Aβ. Moreover, Angiotensin-II and Angiotensin-IV activity have been associated with protection from ischemia via activity at AT2R and possibly AT4R. In addition, Angiotensin-II and Angiotensin-IV activity have been associated with direct effects on memory. Taken together, antihypertensives that increase activity at AT2R and AT4R ("stimulating" antihypertensives) are hypothesized to have greater brain protective effects than those that decrease activity at the same receptors ("inhibiting" antihypertensives). Furthermore, animal data suggest that ARBs could provide greater brain protective effects via activation of MasR via Angiotensin-(1-7), a by-product of Angiotensin II. Less is known about how other antihypertensives affect MasR activation. The relative contributions of antihypertensive-related AT2R, AT4R, and MasR activation on brain effects remain to be elucidated. Angiotensin II receptor type 2 and 4-stimulating antihypertensives defined as ARBs, DiCCBs, and thiazide diuretics. Angiotensin II receptor type 2 and 4-inhibiting antihypertensives defined as ACEIs, BBs, and non-DiCCBs.

Figure modified from: Dalen JW, Marcum ZA*, Gray SL, Barthold D, Moll van Charante EP, van Gool WA, Crane PK, Larson EB, Richard E. Association of angiotensin-II stimulating antihypertensive use and dementia risk: Post hoc analysis of the preDIVA trial. *Neurology* 2021;96:e67-e80.
*co-first author

© 2022 Marcum ZA et al. *JAMA Network Open.*

eMethods 1. SPRINT Treatment Protocol Summary

The SPRINT treatment protocol was flexible in terms of choice and dose of antihypertensive medications, with preferences among the drug classes specified based on cardiovascular disease outcome trials and current guidelines. One or more medications from the following classes were provided by the study for blood pressure management: angiotensin converting enzyme (ACE) inhibitors, angiotensin-II receptor blockers (ARBs), direct vasodilators, thiazide diuretics, loop diuretics, potassium-sparing diuretics, beta-blockers, calcium channel blockers, alpha 1 receptor blockers, and sympatholytics. For both randomized groups, routine visit frequency was monthly for the first 3 months after randomization, then every 3 months for the duration of the trial. By the 6-month visit, most participants were receiving at least one antihypertensive. Participants received medication supplies at regularly scheduled visits in sufficient quantity to last until the next scheduled visit.

© 2022 Marcum ZA et al. *JAMA Network Open.*

eFigure 2. Modified therapeutic intensity score across SPRINT follow up, by angiotensin II receptor type 2 and 4-stimulating only and inhibiting only antihypertensive use[a]

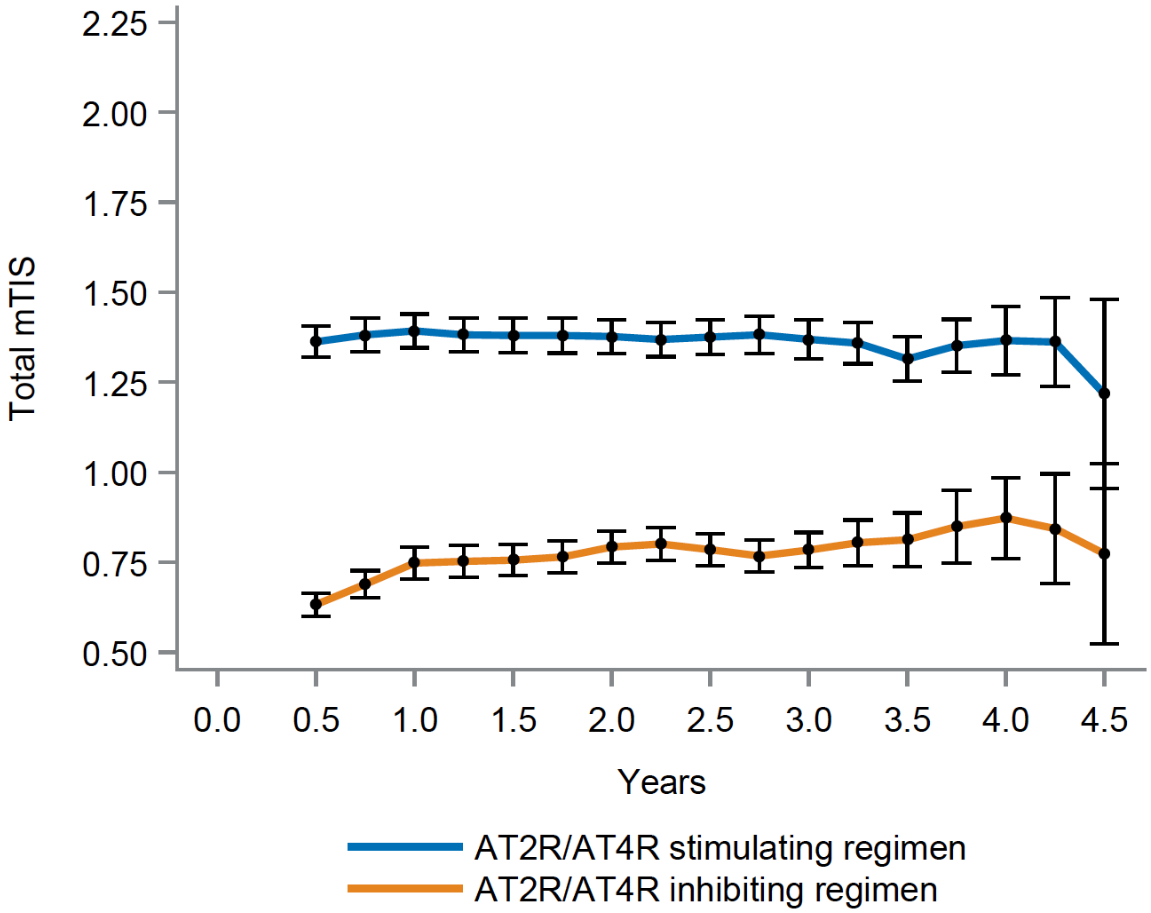

No. with Data

AT2R/AT4R stimulating regimen	2516	2458	2420	2347	2254	1709	1034	476	62
AT2R/AT4R inhibiting regimen	1453	1420	1378	1334	1277	971	572	240	28

[a]The therapeutic intensity score (TIS) is a summary measure that accounts for (1) the number of antihypertensive medications in a patient's regimen; and (2) the dose of each medication that the patient is receiving relative to the Food and Drug Administration (FDA) maximally recommended dose. The modified TIS (mTIS) is therefore defined as below, where n is the number of antihypertensive medications in the regimen, and *i* is each individual antihypertensive medication. For example, a patient taking a regimen of lisinopril 20 mg daily (maximum dose 40 mg) and metoprolol 50 mg (maximum dose 200 mg) would have an mTIS = 0.5+0.25 = 0.75.

$$mTIS = \sum_{i-1}^{n} \frac{Prescribed\ Daily\ Dose_i}{Guideline\text{-}Defined\ Maximum\ Daily\ Dose_i}$$

© 2022 Marcum ZA et al. *JAMA Network Open.*

eFigure 3. Modified therapeutic intensity score across SPRINT follow up, by angiotensin II receptor type 2 and 4-stimulating and inhibiting antihypertensive (“mixed”) use and inhibiting only antihypertensive use[a]

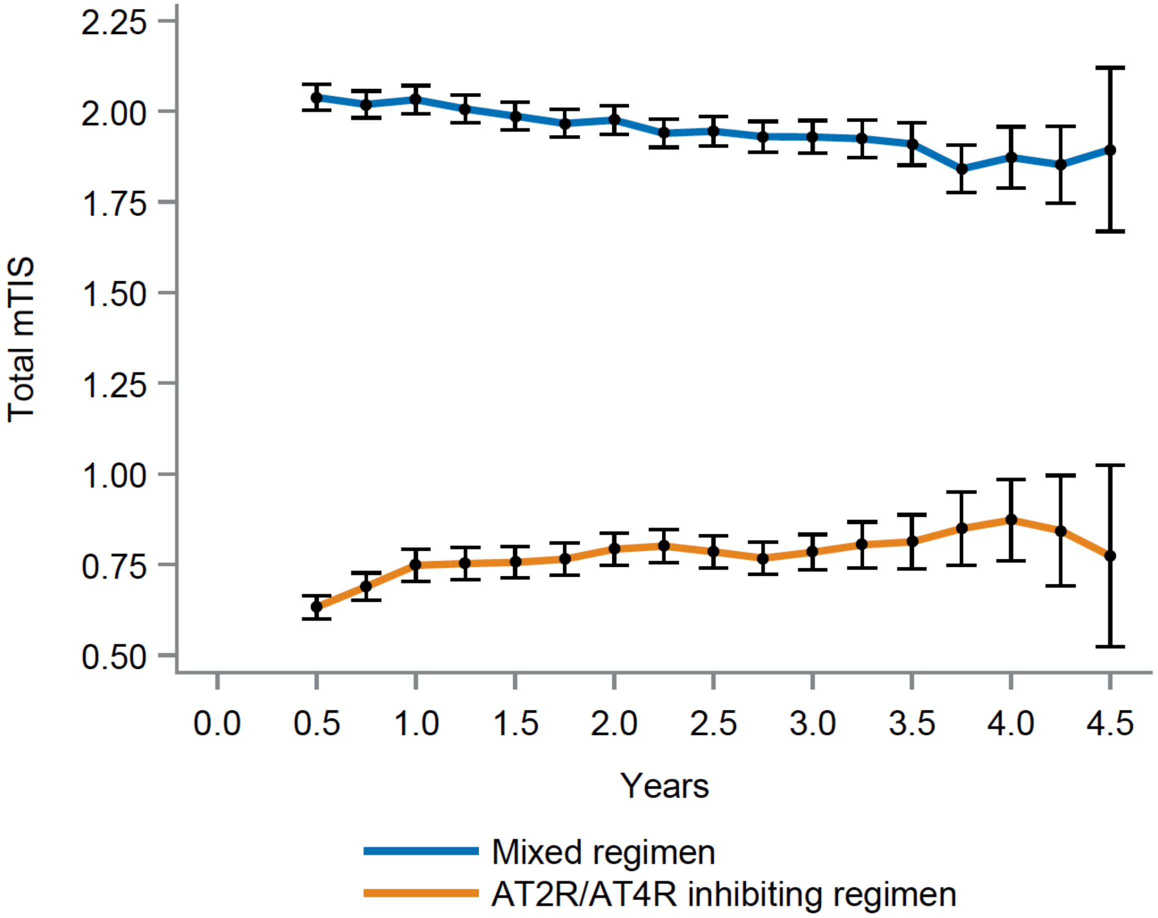

No. with Data

Mixed regimen	4267	4177	4068	4003	3797	2829	1718	772	108
AT2R/AT4R inhibiting regimen	1453	1420	1378	1334	1277	971	572	240	28

[a]The therapeutic intensity score (TIS) is a summary measure that accounts for (1) the number of antihypertensive medications in a patient’s regimen; and (2) the dose of each medication that the patient is receiving relative to the Food and Drug Administration (FDA) maximally recommended dose. The modified TIS (mTIS) is therefore defined as below, where n is the number of antihypertensive medications in the regimen, and *i* is each individual antihypertensive medication. For example, a patient taking a regimen of lisinopril 20 mg daily (maximum dose 40 mg) and metoprolol 50 mg (maximum dose 200 mg) would have an mTIS = 0.5+0.25 = 0.75.

$$mTIS = \sum_{i-1}^{n} \frac{Prescribed\ Daily\ Dose_i}{Guideline\text{–}Defined\ Maximum\ Daily\ Dose_i}$$

© 2022 Marcum ZA et al. *JAMA Network Open.*

eMethods 2. Assessment and adjudication of cognitive outcomes in SPRINT

Ascertaining cognitive status involved 3 steps. First, in-person cognitive screening assessments were administered to all participants at baseline and during follow-up by centrally trained and certified examiners at each local site. Assessments included a test of global cognitive function (Montreal Cognitive Assessment [MoCA]; range, 0-30), learning and memory (Logical Memory forms I and II subtests of the Wechsler Memory Scale; ranges, 0-28 and 0-14), and processing speed (Digit Symbol Coding Test of the Wechsler Adult Intelligence Scale; range, 0-135). For white participants scoring lower than 19 (with <12 years of education) or lower than 21 (with ≥12 years of education) on the MoCA, non-white participants scoring lower than 17 (with <12 years of education) or lower than 19 (with ≥12 years of education) on the MoCA, or any participant with a decrease of 5 or more points from a previous MoCA assessment, a preidentified proxy was administered the Functional Activities Questionnaire, a 10-item measure of functional abilities (range, 0-30).

Second, participants scoring either higher than 0 on the Functional Activities Questionnaire or scoring 1 or lower on the 5-point Delayed Recall subtest of the MoCA underwent further testing using an extended cognitive battery that measured attention/concentration, verbal and nonverbal memory, language, and executive functions. For participants who could not be assessed in persons during follow-up, a validated telephone battery was administered. For participants receiving the telephone battery, the Functional Activities Questionnaire was administered if the participant scored below a preset cut point (≤31) on the Modified Telephone Interview for Cognitive Status. If a participant had died or was otherwise unable to communicate by telephone, the Dementia Questionnaire was administered to a prespecified contact. For all tests and questionnaires, validated Spanish translations were used when available. Otherwise, instruments were translated and then back-translated.

Third, in addition to cognitive test scores and proxy functional status reports, all participants were administered a standardized measure of depressive symptoms, perceived health status, quality of life, current medications, medical problems, and current health habits (smoking, alcohol use, and physical activity). Hospitalizations were also recorded as part of a standardized protocol for ascertainment of serious adverse events with all references to treatment group redacted. These data were reviewed by an expert adjudication panel that included a neurologist, neuropsychologists, geriatricians, and geropsychologists to adjudicate cognitive status. The adjudicators were masked to treatment status. Participants were classified into 1 of 3 primary categories: no cognitive impairment, MCI, or probable dementia. Unclassifiable cases were placed in a “cannot classify” category. Each case was reviewed independently by 2 adjudicators using standardized diagnostic criteria for probable dementia and MCI. Agreements by the 2 adjudicators were final. Disagreements were discussed by the full panel on regularly scheduled conference calls, with the classification decision achieved by a majority vote of the panel members. No subclassification of probable dementia was made. Additional details of the adjudication process can be found in the trial protocol.

© 2022 Marcum ZA et al. *JAMA Network Open.*

eFigure 4. Timeline for follow-up cognitive assessments in SPRINT

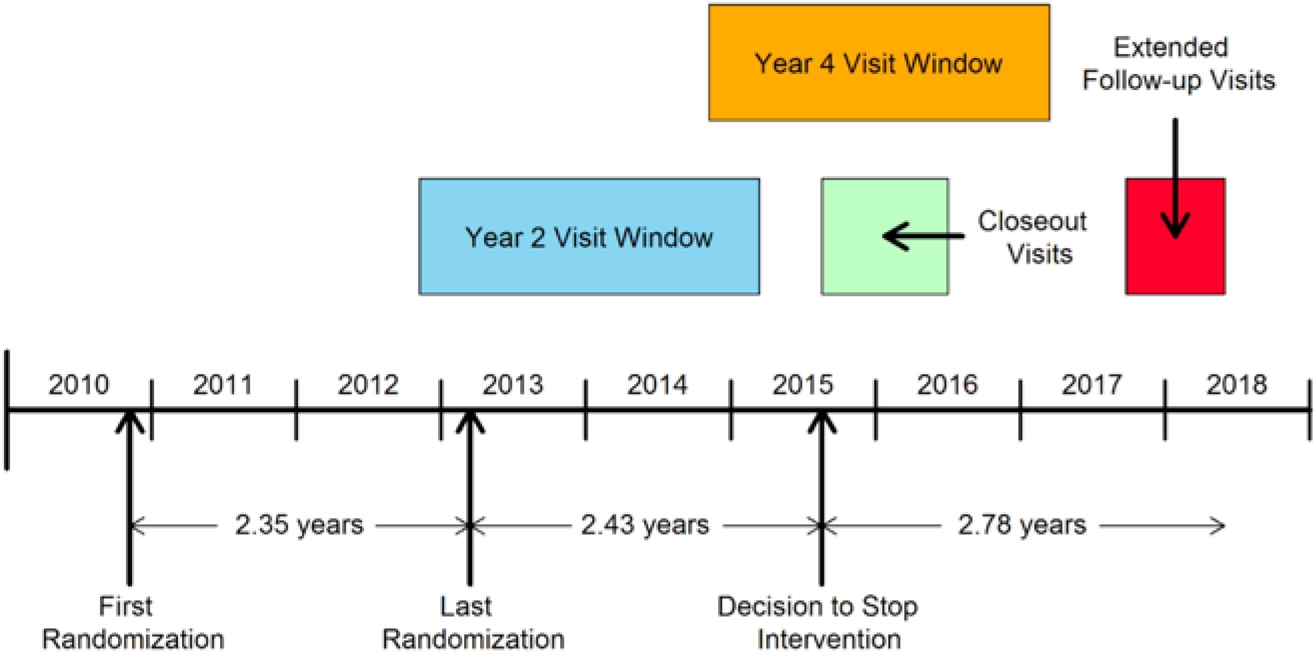

© 2022 Marcum ZA et al. *JAMA Network Open.*

eFigure 5. Distribution of propensity scores, angiotensin II receptor type 2 and 4-stimulating only and inhibiting only antihypertensive use at 6-month visit

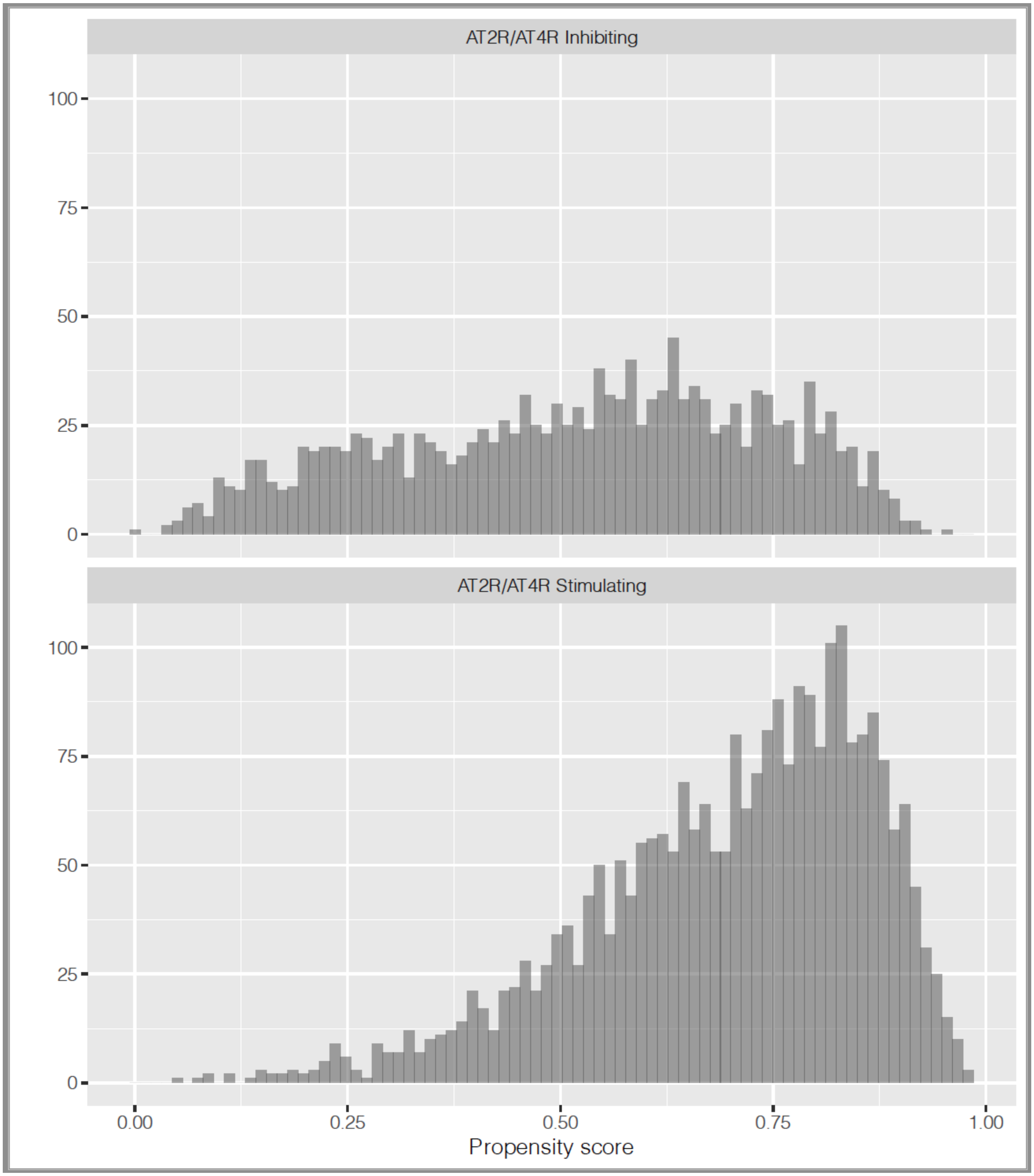

© 2022 Marcum ZA et al. *JAMA Network Open.*

eFigure 6. Distribution of propensity scores, angiotensin II receptor type 2 and 4-stimulating and inhibiting antihypertensive (“mixed”) versus inhibiting only antihypertensive use at 6-month visit

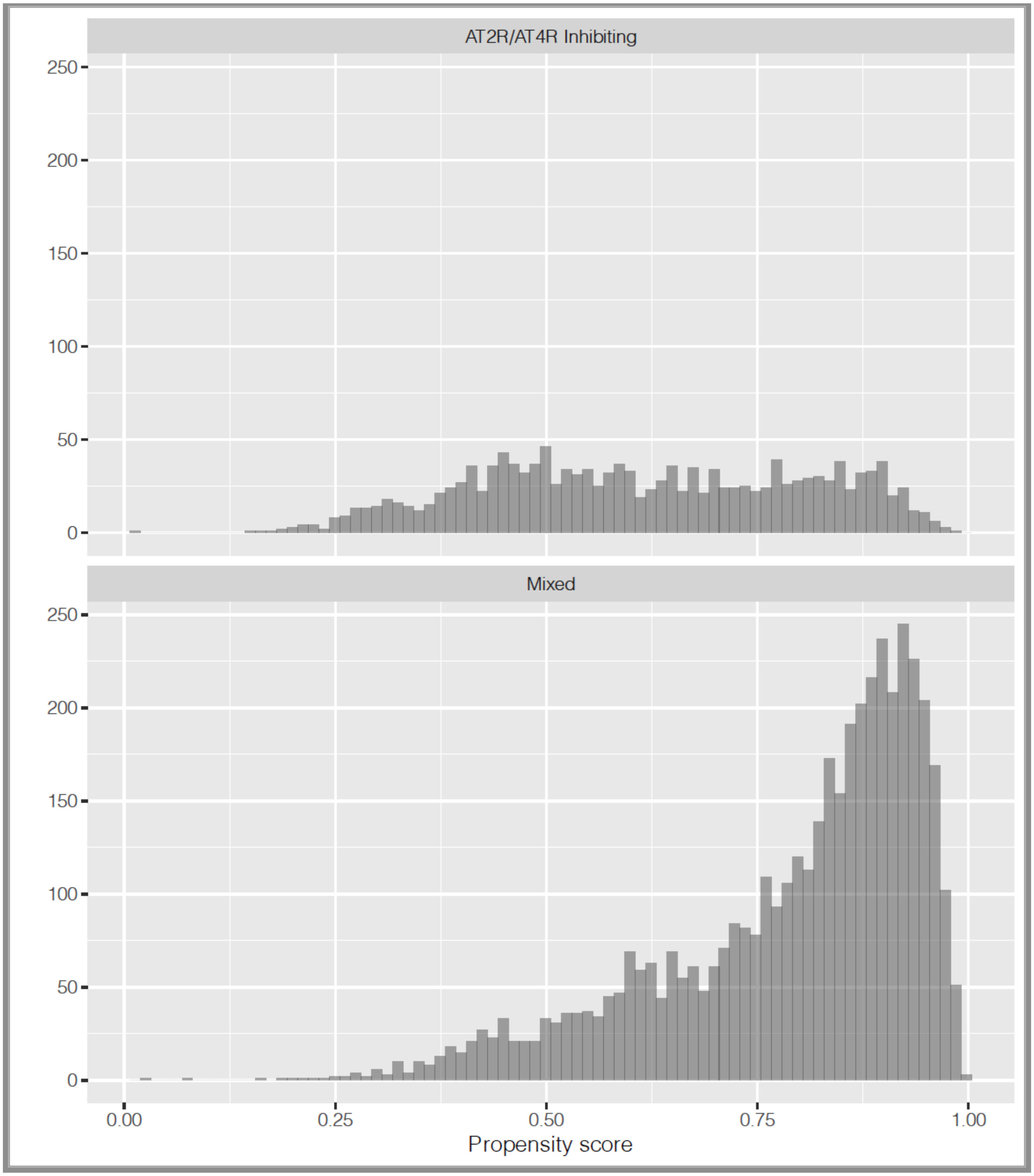

© 2022 Marcum ZA et al. *JAMA Network Open.*

eFigure 7. Absolute standardized differences comparing angiotensin II receptor type 2 and 4-stimulating only and inhibiting only antihypertensive use at 6-month visit, unadjusted and adjusted for average treatment effect

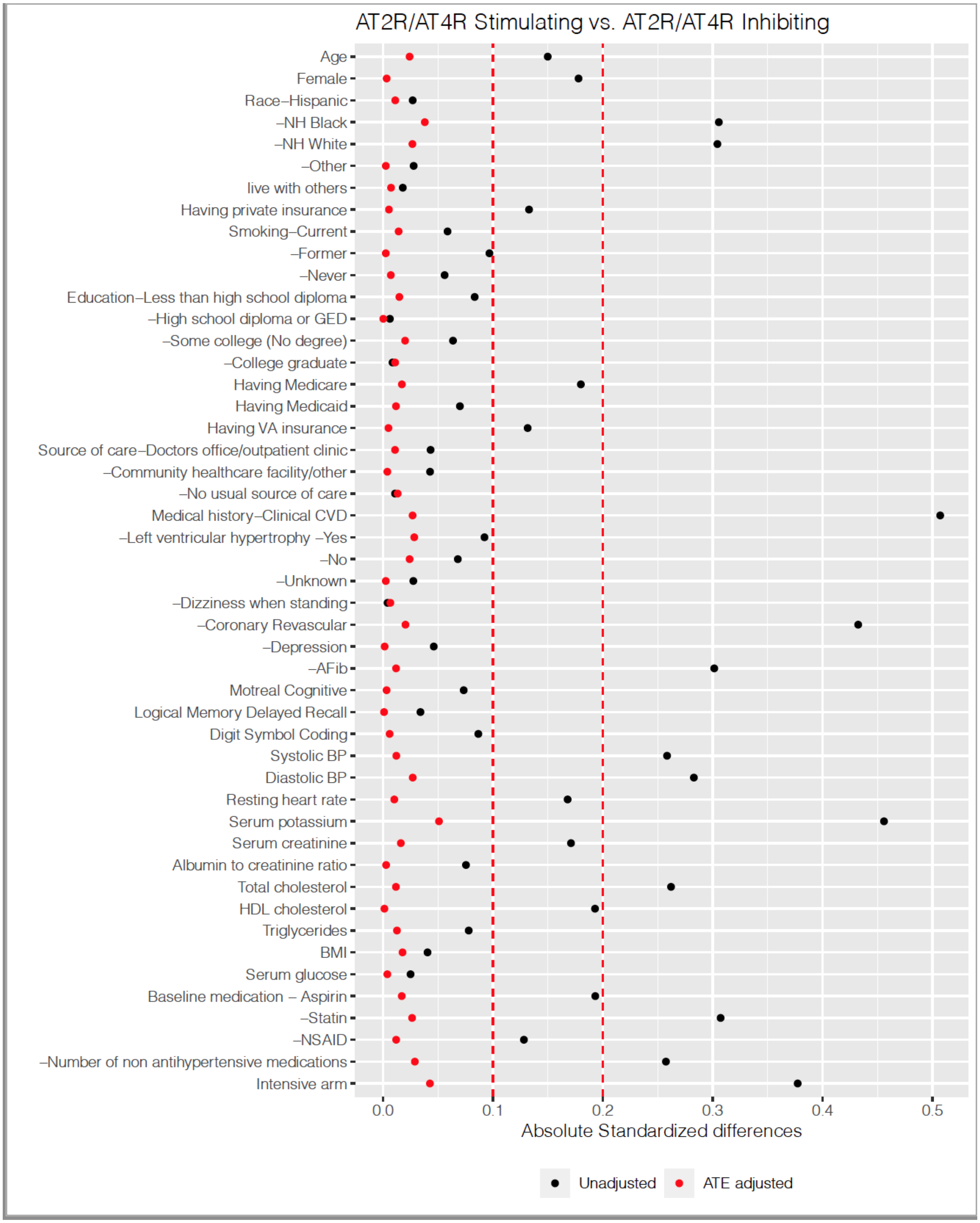

© 2022 Marcum ZA et al. *JAMA Network Open.*

eFigure 8. Systolic blood pressure across SPRINT follow-up, by angiotensin II receptor type 2 and 4-stimulating only and inhibiting only antihypertensive use

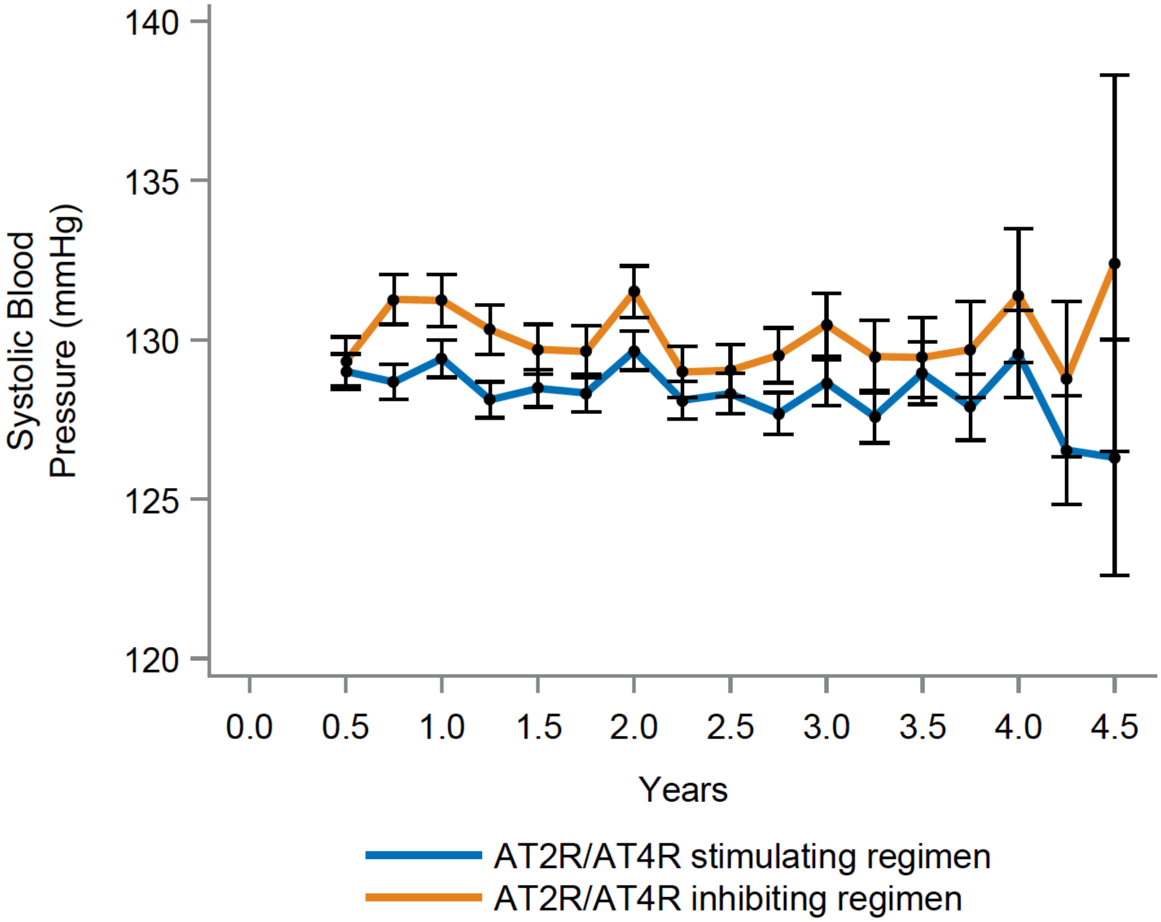

No. with Data

AT2R/AT4R stimulating regimen	2512	2450	2392	2324	2212	1682	1008	471	62
AT2R/AT4R inhibiting regimen	1447	1412	1357	1327	1254	952	558	237	28

© 2022 Marcum ZA et al. *JAMA Network Open.*

eFigure 9. Cumulative incidence curves for secondary outcomes, angiotensin II receptor type 2 and 4-stimulating only versus inhibiting only antihypertensive users

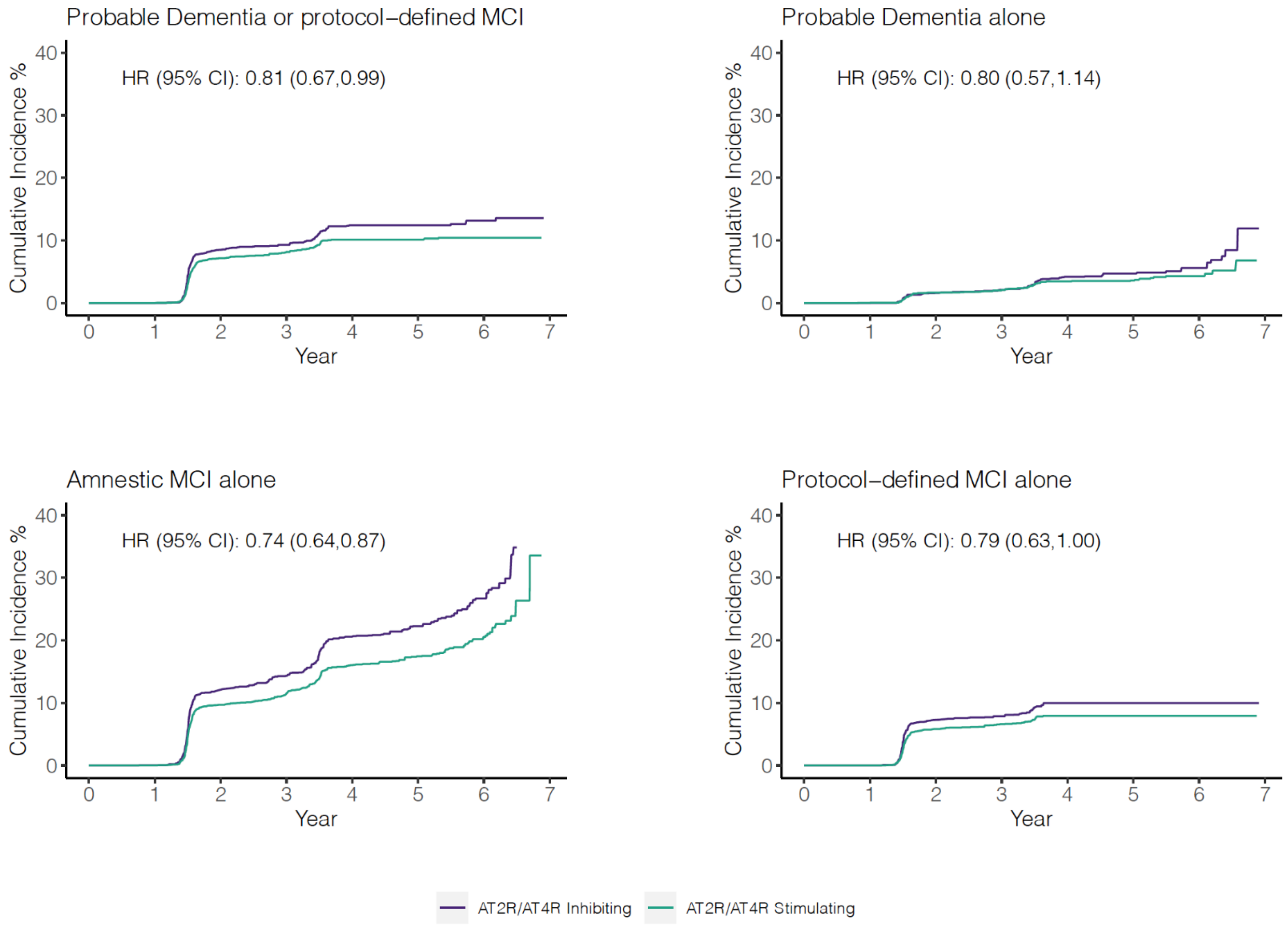

© 2022 Marcum ZA et al. *JAMA Network Open.*

eFigure10. Cumulative incidence curves for secondary outcomes with death as a composite, angiotensin II receptor type 2 and 4-stimulating only versus inhibiting only antihypertensive users

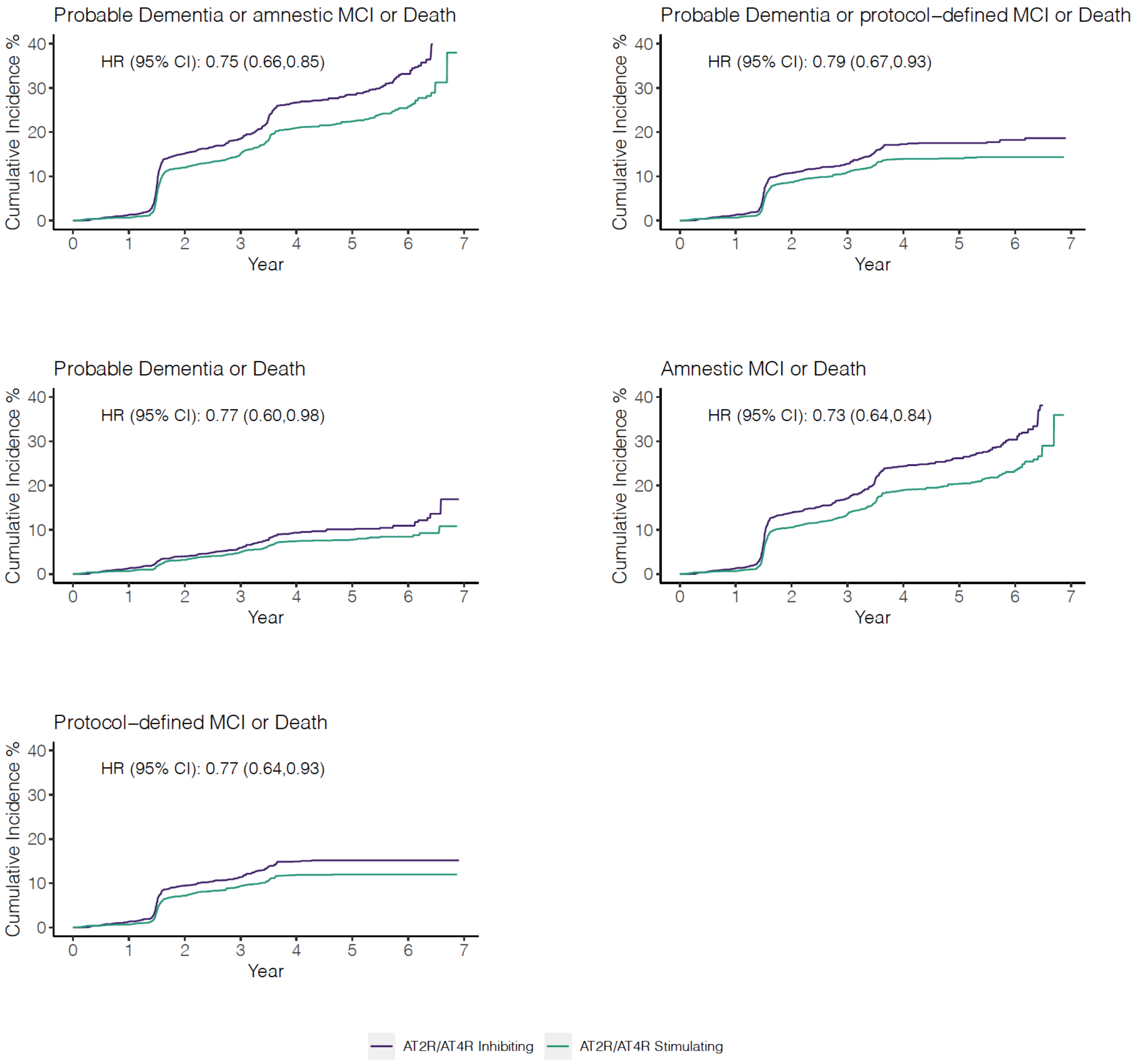

© 2022 Marcum ZA et al. *JAMA Network Open.*

**eTable 1. Baseline characteristics between prevalent users of angiotensin II receptor type 2 and 4-stimulating and inhibiting antihypertensives ("mixed") versus inhibiting alone at 6-month visit, before and after inverse probability weighting**						
	Before weighting			***After weighting***		
**Variable, N (%)**	**Mixed**	**AT2R/AT4R Inhibiting**	**ASD**	**Mixed**	**AT2R/AT4R Inhibiting**	**ASD**
	**(N =4505)**	**(N =1536)**	**--**			**--**
Demographics	--	--	--	--	--	--
Age (years), mean (sd)	68 (9.4)	68.6 (9.2)	0.07	68.2 (9.4)	68.7 (9.4)	0.05
Female	1615 (35.8)	461 (30.0)	0.12	35	35.8	0.02
Male	2890 (64.2)	1075 (70.0)	--	65	64.2	--
Race/Ethnicity	--	--	--	--	--	--
Hispanic	445 (9.9)	171 (11.1)	0.04	10.4	10.8	0.01
Non-Hispanic Black	1418 (31.5)	301 (19.6)	0.26	28.6	25.4	0.07
Non-Hispanic White	2562 (56.9)	1038 (67.6)	0.22	59.2	61.5	0.05
Social and behavioral	--	--	--	--	--	--
Lives with others	3222 (71.5)	1096 (71.4)	0	71.2	70.4	0.02
Has private insurance	1875 (41.6)	608 (39.6)	0.04	41.3	41.5	0.01
Current smoker	620 (13.8)	179 (11.7)	0.06	13.3	12.3	0.03
Former smoker	1987 (44.1)	678 (44.1)	0	44	43.5	0.01
Never smoker	1894 (42.0)	677 (44.1)	0.04	42.7	44.2	0.03
Education	--	--	--	--	--	--
Less than high school	450 (10.0)	164 (10.7)	0.02	10.2	11.1	0.03
High school graduate only	784 (17.4)	236 (15.4)	0.05	16.8	15.8	0.03
Post high school graduate	1606 (35.6)	511 (33.3)	0.05	35.1	33.9	0.03
College graduate or greater	1665 (37.0)	625 (40.7)	0.08	37.9	39.3	0.03
Health insurance status	--	--	--	--	--	--
Medicare	2501 (55.5)	926 (60.3)	0.1	57	59.4	0.05
Medicaid	329 (7.3)	123 (8.0)	0.03	7.5	8.2	0.03
VA	963 (21.4)	333 (21.7)	0.01	21.4	20.8	0.01
Usual source of care	--	--	--	--	--	--
Doctor's office/outpatient clinic	3817 (84.7)	1324 (86.2)	0.04	85.3	86.4	0.03
Community healthcare facility/other	519 (11.5)	148 (9.6)	0.06	11.1	10.6	0.02
No usual source of care	161 (3.6)	62 (4.0)	0.02	3.6	3	0.03
Medical history	--	--	--	--	--	--
Clinical CVD	828 (18.4)	372 (24.2)	0.14	19.6	20.3	0.02
Left ventricular hypertrophy	973 (21.6)	215 (14.0)	0.19	19.7	17.4	0.06
Dizziness when standing	173 (3.8)	73 (4.8)	0.04	4.1	4.1	0
History of coronary revascularization	510 (11.3)	231 (15.0)	0.11	12.1	12.3	0.01
History of depression	828 (18.4)	296 (19.3)	0.02	18.5	18.9	0.01
History of atrial fibrillation or flutter	419 (9.3)	180 (11.7)	0.08	10	10.7	0.02
Baseline cognitive assessments	--	--	--	--	--	--
Montreal Cognitive Assessment[a], median (IQR)	22.8 (4.1)	22.8 (4.2)	0.01	22.8 (4.1)	22.7 (4.3)	0.01
Logical Memory form II[b], median (IQR)	8 (3.4)	8.3 (3.3)	0.06	8.1 (3.4)	8 (3.4)	0.01
Digit Symbol Coding Test[c], median (IQR)	50.2 (15.1)	50.7 (15)	0.03	50.2 (15.1)	50 (15.3)	0.01
Clinical/laboratory measurements	--	--	--	--	--	--
Systolic BP (mm Hg), mean (sd)	141.6 (16.2)	135.8 (15)	0.36	140.2 (16.1)	139.8 (15.8)	0.03

© 2022 Marcum ZA et al. *JAMA Network Open.*

	Before weighting			After weighting		
**Variable**	**Mixed**	**AT2R/AT4R Inhibiting**	**ASD**	**Mixed**	**AT2R/AT4R Inhibiting**	**ASD**
Diastolic BP (mm Hg), mean (sd)	78.3 (12.4)	76.1 (11.2)	0.18	77.7 (12.4)	77.1 (11.4)	0.05
Resting heart rate (beats/minute), mean (sd)	65.4 (11.7)	65.8 (11.4)	0.03	65.5 (11.8)	65.4 (11.2)	0
Serum potassium (mEq/L), mean (sd)	4.2 (0.4)	4.3 (0.5)	0.35	4.2 (0.6)	4.3 (0.4)	0.05
Serum creatinine (mg/dL), mean (sd)	1.1 (0.4)	1.1 (0.3)	0.01	1.1 (0.4)	1.1 (0.4)	0.02
Albumin to creatinine ratio (mg/g), mean (sd)	49.6 (179.9)	42.8 (184.8)	0.04	48 (176.3)	49 (206.9)	0.01
Total cholesterol (mg/dL), mean (sd)	188.7 (41.5)	184.3 (40.8)	0.11	187.8 (41.3)	186.5 (40.5)	0.03
HDL cholesterol (mg/dL), mean (sd)	52.3 (14.5)	51.6 (14.3)	0.05	52.2 (14.4)	52.4 (14.3)	0.01
Triglycerides (mg/dL), mean (sd)	129.6 (92.3)	127.7 (77.1)	0.02	128.8 (89)	126.7 (77.7)	0.02
Body mass index (kg/m^2), mean (sd)	30 (5.8)	29.6 (5.7)	0.07	29.9 (5.8)	29.7 (6.1)	0.02
Serum glucose (mg/dL), mean (sd)	99.5 (13.5)	98.4 (13.1)	0.09	99.1 (13.3)	98.6 (14.9)	0.03
Medication use	--	--	--	--	--	--
Aspirin	2522 (56.0)	867 (56.4)	0	56.1	56.4	0.01
Statin	2098 (46.6)	777 (50.6)	0.08	47.5	48.3	0.02
NSAID	1823 (40.5)	614 (40.0)	0.01	40.3	39.7	0.01
Number of non-antihypertensive medications	3.9 (3.2)	4.1 (3.2)	0.06	3.9 (3.3)	4 (3.3)	0.03
Randomized to intensive treatment	2866 (63.6)	439 (28.6)	0.71	55.1	52.7	0.05

[a]Scores range from 0 to 30, with higher scores denoting better global cognitive function.
[b]Subtest of the Wechsler Memory Scale to measure learning and memory skills. Scores range from 0 to 14, with higher scores denoting better cognitive function.
[c]Subtest of the Wechsler Adult Intelligence Scale to measure processing speed. Scores range from 0 to 135, with higher scores denoting better cognitive function.

ASD: absolute standardized difference; BP: blood pressure; AT2R/AT4R: Angiotensin II receptor type 2 and 4; CVD: cardiovascular disease; HDL: high-density lipoprotein; NSAID: non-steroidal anti-inflammatory drug; sd: standard deviation; VA: Veterans Affairs

Missing values (mixed/inhibiting): lives with others=1/2, smoking=4/2, source of care=8/2, left ventricular hypertrophy =232/86, dizziness when standing=8/3, depression=3/2, Montreal Cognitive Assessment=28/11, Logical Memory Delayed Recall=35/14, Digit Symbol Coding=42/17, resting heart rate=1/1, serum potassium=5/1, serum creatinine=22/3, albumin to creatinine ratio=187/75, total cholesterol=11/4, HDL cholesterol=11/4, triglycerides=11/4, BMI=26/12, serum glucose=11/4.

Mixed defined as use of both angiotensin II receptor type 2 and 4-stimulating and inhibiting antihypertensives. Angiotensin II receptor type 2 and 4-stimulating antihypertensives defined as use of angiotensin II receptor blockers, dihydropyridine calcium channel blockers, and/or thiazides. Angiotensin II receptor type 2 and 4-inhibiting antihypertensives defined as use of angiotensin-converting enzyme inhibitors, ß-blockers, and/or non-dihydropyridine calcium channel blockers.

© 2022 Marcum ZA et al. *JAMA Network Open.*

eFigure 11. Absolute standardized differences comparing angiotensin II receptor type 2 and 4-stimulating and inhibiting (“mixed) versus inhibiting only antihypertensive use at 6-month visit, unadjusted and adjusted for average treatment effect

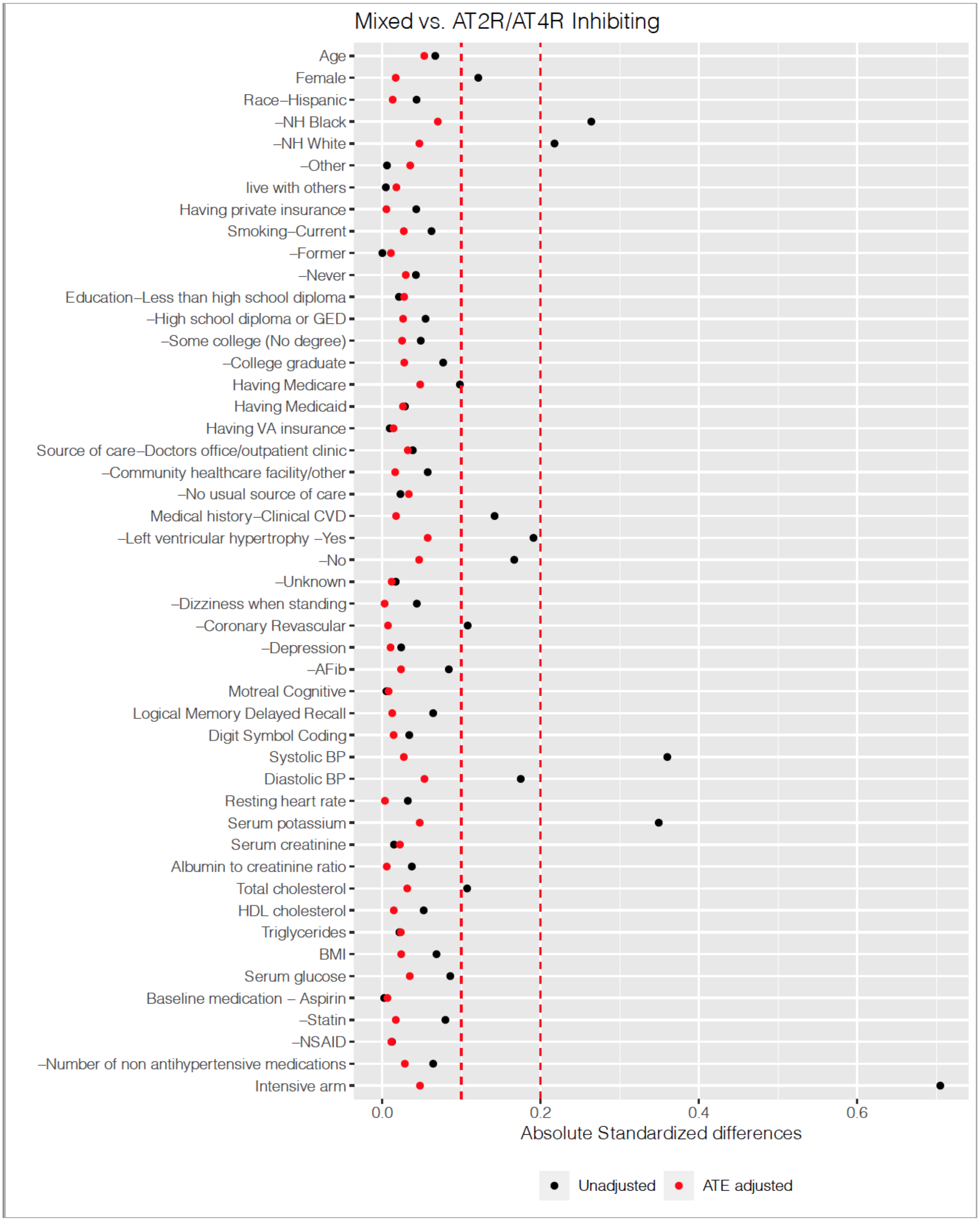

© 2022 Marcum ZA et al. *JAMA Network Open.*

eFigure 12. Systolic blood pressure across SPRINT follow up, by angiotensin II receptor type 2 and 4-stimulating and inhibiting (“mixed”[a]) and inhibiting only antihypertensive use

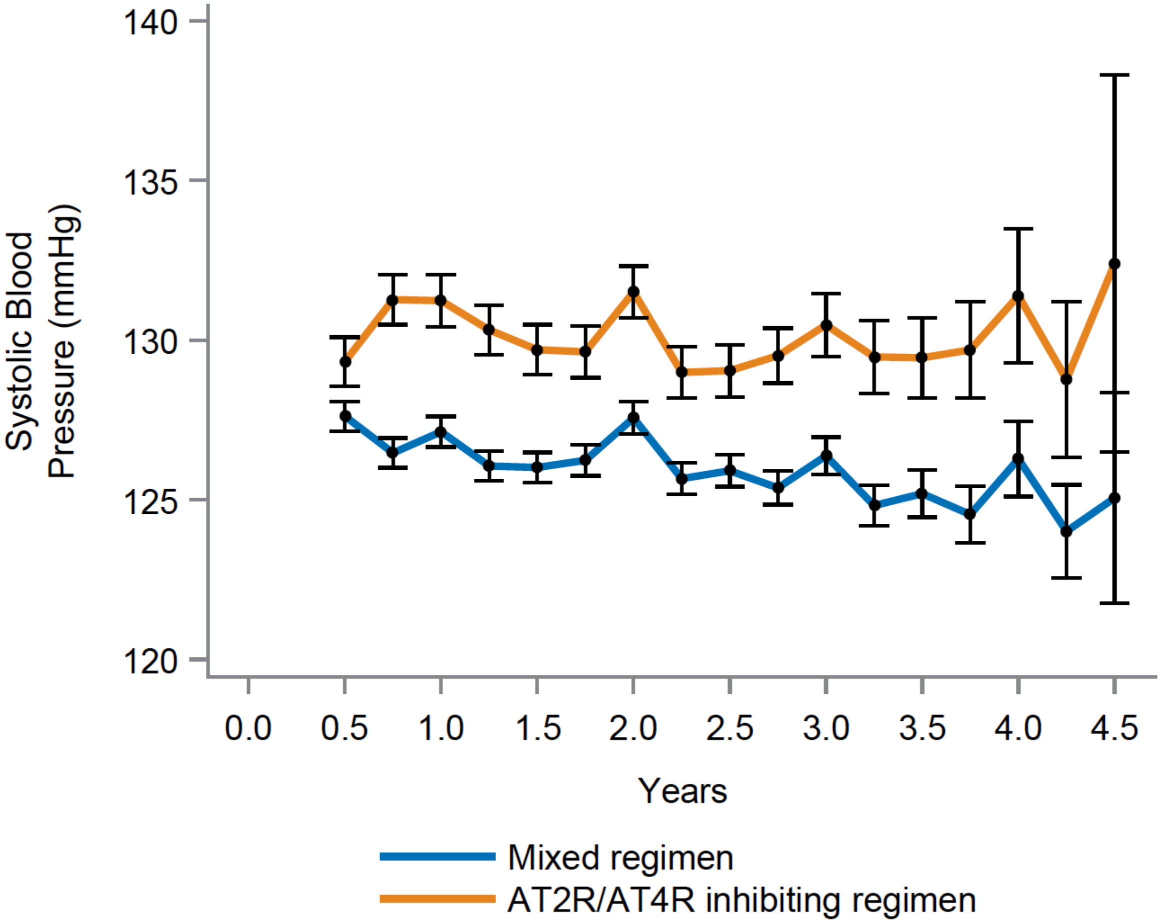

**No. with Data**									
Mixed regimen	4251	4152	4003	3955	3727	2788	1683	761	108
AT2R/AT4R inhibiting regimen	1447	1412	1357	1327	1254	952	558	237	28

[a]Mixed antihypertensive regimen required use of at least one angiotensin II receptor type 2 and 4-stimulating and at least one inhibiting antihypertensive, resulting in a lower systolic blood pressure over SPRINT follow up.

© 2022 Marcum ZA et al. *JAMA Network Open.*

eTable 2. Inverse probability treatment weighting-adjusted incidence rates and hazard ratios for primary and secondary outcomes, angiotensin II receptor type 2 and 4-stimulating and inhibiting ("mixed") versus inhibiting only antihypertensive users

	No. events (events per 1000 person years)		HR (95% CI)
**Outcome**	**Mixed users (N=4,505)**	**AT2R/AT4R4 Inhibiting only users (N=1,536)**	**Mixed vs. AT2R/AT4R4 inhibiting**
**Primary outcome (censoring death)**	-	-	-
Probable dementia or amnestic MCI[a]	859 (50)	355 (62)	0.80 (0.71-0.91)
**Secondary outcomes (censoring death)**	-	-	-
Probable dementia alone	165 (9)	67 (11)	0.90 (0.66-1.24)
Amnestic MCI alone	738 (44)	312 (56)	0.79 (0.69-0.91)
Protocol-defined MCI alone	297 (18)	141 (22)	0.79 (0.63-0.98)
Probable dementia or protocol-defined MCI	417 (24)	183 (29)	0.82 (0.68-0.99)
**Composite outcome (incorporating death)**	-	-	-
Probable dementia or amnestic MCI or death	1050 (61)	428 (77)	0.80 (0.71-0.89)
Probable dementia or death	395 (22)	150 (26)	0.86 (0.69-1.06)
Amnestic MCI or death	945 (55)	389 (69)	0.79 (0.70-0.90)
Protocol-defined MCI or death	536 (31)	226 (38)	0.82 (0.69-0.97)
Probable dementia or protocol-defined MCI or death	640 (37)	264 (45)	0.82 (0.70-0.96)
Death	250 (18)	90 (21)	0.85 (0.65-1.12)

AT2R/AT4R: Angiotensin II receptor type 2 and 4; CI: confidence interval; HR: hazard ratio; MCI: mild cognitive impairment

Mixed defined as use of both angiotensin II receptor type 2 and 4-stimulating and inhibiting antihypertensives. Angiotensin II receptor type 2 and 4-stimulating antihypertensives defined as use of angiotensin II receptor blockers, dihydropyridine calcium channel blockers, and/or thiazides. Angiotensin II receptor type 2 and 4-inhibiting antihypertensives defined as use of angiotensin-converting enzyme inhibitors, ß-blockers, and/or non-dihydropyridine calcium channel blockers.

[a]Median follow up time: 4.7 years (95% CI, 4.7-4.8)

© 2022 Marcum ZA et al. *JAMA Network Open.*

eTable 3. Incidence rates and hazard ratios comparing the association between angiotensin II receptor type 2 and 4-stimulating and inhibiting ("mixed") versus inhibiting only antihypertensive users and probable dementia or amnestic mild cognitive impairment, by covariate adjustment strategy and among sub-groups

Method	N in model	No. events (events per 1000 person years) Mixed users	No. events (events per 1000 person years) AT2R/AT4R4 Inhibiting only users	Mixed vs. AT2R/AT4R4 inhibiting HR (95% CI)
**Covariate adjustment strategy**				
IPW adjusted (primary analysis)	5996	859 (50)	355 (62)	0.80 (0.71-0.91)
Unadjusted	5996	859 (50)	355 (62)	0.82 (0.72-0.92)
Minimally adjusted (age, sex, race)	5996	859 (50)	355 (62)	0.78 (0.69-0.88)
Multivariable adjusted	5996	859 (50)	355 (62)	0.79 (0.69-0.92)
Propensity score as covariate	5996	859 (50)	355 (62)	0.81 (0.72-0.91)
Propensity score stratification	5996	859 (50)	355 (62)	0.81 (0.71-0.93)
Propensity score matching	3044	300 (53)	355 (62)	0.85 (0.70-1.04)
Matching weight adjusted[a]	5996	859 (51)	355 (62)	0.82 (0.72-0.93)
**Sub-group analyses**				
Age	5996	-	-	-
<75 years	4254	448 (34)	168 (39)	0.87 (0.72-1.05)
≥75 years	1744	411 (97)	187 (127)	0.76 (0.64-0.91)
P for interaction	-	-	-	*P* = 0.34
Sex	5996	-	-	-
Female	2067	283 (46)	101 (58)	0.77 (0.62-0.96)
Male	3931	576 (52)	254 (64)	0.82 (0.70--0.96)
P for interaction	-	-	-	*P* = 0.68
Race/Ethnicity	5996	-	-	-
Hispanic	615	91 (60)	55 (78)	0.73 (0.48-1.11)
Non-Hispanic Black	1708	315 (61)	79 (76)	0.80 (0.61-1.04)
Non-Hispanic White	3569	431 (43)	213 (52)	0.82 (0.69-0.96)
P for interaction	-	-	-	*P* = 0.90
Clinical cardiovascular disease	5996	-	-	-
Yes	1189	191 (61)	79 (72)	0.85 (0.63-1.13)
No	4809	668 (47)	276 (60)	0.78 (0.68-0.90)
P for interaction	-	-	-	*P* = 0.64
Renal impairment	5996	-	-	-
Estimated GFR <60 mL/min/1.73m^2	1862	348 (68)	121 (80)	0.84 (0.68-1.05)
Estimated GFR ≥60 mL/min/1.73m^2	4134	511 (42)	234 (54)	0.79 (0.68-0.92)
P for interaction	-	-	-	*P* = 0.64
BMI	5996	-	-	-
<25	1122	197 (69)	94 (81)	0.86 (0.67-1.10)
25 to <30	2329	333 (48)	139 (65)	0.73 (0.59-0.90)
≥30	2547	329 (43)	122 (51)	0.85 (0.68-1.05)
P for interaction	-	-	-	*P* = 0.53
No. antihypertensive medications	5996	-	-	-
2	2012	267 (45)	103 (55)	0.82 (0.65-1.02)
3	1894	338 (52)	37 (79)	0.65 (0.44-0.96)
4	1179	254 (60)	7 (77)	0.76 (0.21-2.67)
P for interaction	-	-	-	*P* = 0.61
Systolic blood pressure	5996	-	-	-
Tertile 1 (72-133 mmHg)	2120	266 (46)	141 (64)	0.71 (0.58-0.87)
Tertile 2 (134-145 mmHg)	1879	235 (43)	120 (57)	0.74 (0.59-0.93)
Tertile 3 (146-231 mmHg)	1999	358 (62)	94 (59)	1.06 (0.84-1.36)
P for interaction	-	-	-	*P* = 0.03
SPRINT randomization arm	5996	-	-	-
Intensive arm	3289	524 (47)	98 (53)	0.88 (0.71-1.09)
Standard arm	2709	335 (53)	257 (66)	0.81 (0.70-0.94)
P for interaction	-	-	-	*P* = 0.53

AT2R/AT4R: Angiotensin II receptor type 2 and 4; BMI: body mass index; CI: confidence interval; GFR: glomerular filtration rate; HR: hazard ratio; IPW: inverse probability weighted

© 2022 Marcum ZA et al. *JAMA Network Open.*

Mixed defined as use of both angiotensin II receptor type 2 and 4-stimulating and inhibiting antihypertensives. Angiotensin II receptor type 2 and 4-stimulating antihypertensives defined as use of angiotensin II receptor blockers, dihydropyridine calcium channel blockers, and/or thiazides. Angiotensin II receptor type 2 and 4-inhibiting antihypertensives defined as use of angiotensin-converting enzyme inhibitors, ß-blockers, and/or non-dihydropyridine calcium channel blockers.
[a]The same PS model from primary analysis was used to estimate matching weights, which provides a weighted analogue to 1:1 paired PS matching.

eFigure 13. Cumulative incidence curves for probable dementia or amnestic mild cognitive impairment, angiotensin II receptor type 2 and 4-stimulating and inhibiting (“mixed”) versus inhibiting only antihypertensive users

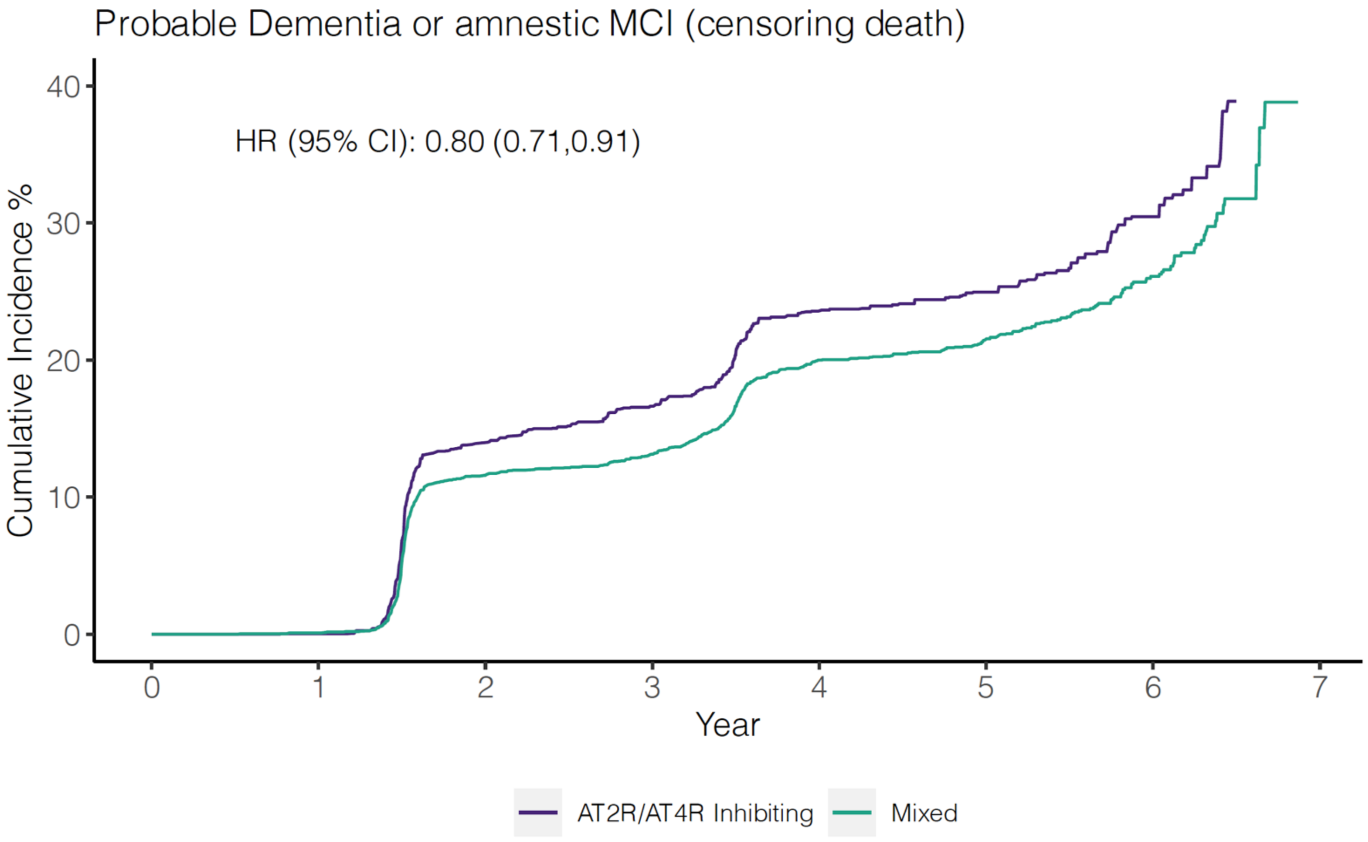

© 2022 Marcum ZA et al. *JAMA Network Open.*

eTable 4. Inverse probability treatment weighting-adjusted incidence rates and hazard ratios for negative control outcomes, angiotensin II receptor type 2 and 4-stimulating only versus inhibiting only antihypertensive use

	No. events (events per 1000 person years)		HR (95% CI)
**Negative control outcome**	**AT2R/AT4R4 Stimulating only users (N=2,644)**	**AT2R/AT4R4 Inhibiting only users (N=1,536)**	**AT2R/AT4R4 Stimulating vs. AT2R/AT4R4 inhibiting**
Infectious negative control outcome[a]	90 (12)	77 (16)	0.78 (0.55-1.11)
Orthopedic negative control outcome[b]	102 (13)	77 (18)	0.72 (0.52-1.00)
Hematologic negative control outcome[c]	48 (7)	40 (9)	0.83 (0.50-1.37)

Numbers in table are expressed as frequency of event (rate per 1000 person-years).
AT2R/AT4R: Angiotensin II receptor type 2 and 4; CI: confidence interval; HR: hazard ratio.

We carefully reviewed the list of serious adverse events and identified negative control outcomes that are unlikely to be causally related to antihypertensive use.

[a]Infectious negative control outcome: Composite of pneumonia, urinary tract infection, sepsis, cellulitis
[b]Orthopedic negative control outcome: Composite of knee arthroplasty, spinal fusion surgery, spinal laminectomy, osteoarthritis
[c]Hematologic negative control outcome: Composite of pulmonary embolism, deep vein thrombosis, gastrointestinal hemorrhage

© 2022 Marcum ZA et al. *JAMA Network Open.*

eTable 5. Inverse probability treatment weighting-adjusted incidence rates and hazard ratios for negative control outcomes, angiotensin II receptor type 2 and 4-stimulating and inhibiting ("mixed") versus inhibiting only antihypertensive use

	No. events (events per 1000 person years)		HR (95% CI)
**Negative control outcome**	**Mixed users (N=4,505)**	**AT2R/AT4R4 Inhibiting only users (N=1,536)**	**Mixed vs. AT2R/AT4R4 inhibiting**
Infectious negative control outcome[a]	210 (16)	77 (20)	0.80 (0.59-1.09)
Orthopedic negative control outcome[b]	206 (15)	77 (18)	0.83 (0.61-1.15)
Hematologic negative control outcome[c]	81 (6)	40 (11)	0.57 (0.37-0.88)

Numbers in table are expressed as frequency of event (rate per 1000 person-years).
AT2R/AT4R: Angiotensin II receptor type 2 and 4; CI: confidence interval; HR: hazard ratio.

We carefully reviewed the list of serious adverse events and identified negative control outcomes that are unlikely to be causally related to antihypertensive use.

[a]Infectious negative control outcome: Composite of pneumonia, urinary tract infection, sepsis, cellulitis
[b]Orthopedic negative control outcome: Composite of knee arthroplasty, spinal fusion surgery, spinal laminectomy, osteoarthritis
[c]Hematologic negative control outcome: Composite of pulmonary embolism, deep vein thrombosis, gastrointestinal hemorrhage

© 2022 Marcum ZA et al. *JAMA Network Open.*

eTable 6. Antihypertensive sub-class use at SPRINT randomization and 6-month visit, defined by angiotensin II receptor type 2 and 4-stimulating and inhibiting classification at 6-month visit

	Regimen at 6-month visit					
**Antihypertensive sub-classes**	**AT2R/AT4R4 Inhibiting N=1536**		**AT2R/AT4R4 Stimulating N=2644**		**Mixed N=4505**	
	**SPRINT randomization**	**6-month visit**	**SPRINT randomization**	**6-month visit**	**SPRINT randomization**	**6-month visit**
**AT2R/AT4R4 Inhibiting antihypertensives, N (%)**	-	-	-	-	-	-
ACE-I	878 (57.2)	1068 (70)	302 (11.4)	0 (0)	2180 (48.4)	2600 (58)
Non-DHP CCB	112 (7.3)	125 (8)	56 (2.1)	0 (0)	321 (7.1)	336 (7)
Beta blocker	721 (46.9)	775 (50)	199 (7.5)	0 (0)	2366 (52.5)	2674 (59)
**AT2R/AT4R4 Stimulating antihypertensives, N (%)**	-	-	-	-	-	-
ARB	58 (3.8)	0 (0)	1052 (39.8)	1529 (58)	829 (18.4)	1149 (26)
DHP CCB	162 (10.5)	0 (0)	925 (35.0)	1378 (52)	1620 (36.0)	2356 (52)
Thiazide diuretic	312 (20.3)	0 (0)	1243 (47.0)	1626 (61)	2031 (45.1)	3043 (68)
**Other antihypertensives, N (%)**	-	-	-	-	-	-
Loop diuretic	146 (9.5)	174 (11)	78 (3.0)	89 (3)	290 (6.4)	301 (7)
Alpha1 blocker	198 (12.9)	105 (7)	230 (8.7)	166 (6)	446 (9.9)	284 (6)
Miscellaneous	120 (7.8)	102 (7)	293 (11.1)	295 (11)	487 (10.8)	600 (13)

AT2R/AT4R: Angiotensin II receptor type 2 and 4; ACE-I: angiotensin converting enzyme inhibitor; ARB: angiotensin-II receptor blocker; CCB: calcium channel blocker; DHP: dihydropyridine

© 2022 Marcum ZA et al. *JAMA Network Open.*
